# National school food standards in England: a cross-sectional study to explore compliance in secondary schools and impact on pupil nutritional intake

**DOI:** 10.1186/s12966-024-01672-w

**Published:** 2024-10-24

**Authors:** Miranda Pallan, Marie Murphy, Breanna Morrison, Alice Sitch, Ashley Adamson, Suzanne Bartington, Alexandra Dobell, Rhona Duff, Emma Frew, Tania Griffin, Kiya Hurley, Emma Lancashire, Louise McLeman, Sandra Passmore, Irina Pokhilenko, Maisie Rowland, Vahid Ravaghi, Suzanne Spence, Peymane Adab

**Affiliations:** 1 Department of Applied Health Sciences, Murray learning Centre, University of Birmingham, Edgbaston, Birmingham, B15 2TT UK; 2 NIHR Birmingham Biomedical Research Centre, Institute of Translational Medicine, University Hospitals Birmingham NHS Foundation Trust, University of Birmingham, Birmingham, B15 2TH UK; 3 Population Health Sciences Institute, Faculty of Medical Sciences, Newcastle University, M1.151 William Leech Building, Framlington Place, Newcastle upon Tyne, NE2 4HH UK; 4 Department for Health, University of Bath, 1 West, Claverton Down, Bath, BA2 7AY UK; 5 Services for Education, Unit 3 Holt Court, Holt Street, Birmingham, B7 4AX UK; 6 The School of Dentistry, University of Birmingham, 5 Mill Pool Way, Edgbaston, Birmingham, B5 7EG UK

**Keywords:** Schools, Adolescents, Food, Nutrition

## Abstract

**Background:**

Many countries have introduced school food standards to improve the dietary intakes of school-aged children. England has school food standards (SFS) legislation in place but little is known about how well secondary schools comply with this. We aimed to assess compliance with the SFS legislation in English secondary schools and explore the impact of the SFS on pupils’ nutritional intake.

**Methods:**

We conducted a cross-sectional study with English secondary schools from 2019 to 2022. We compared SFS compliance and pupil nutritional intake in schools mandated or not mandated to comply with the SFS legislation, and explored the association between school compliance and pupil nutritional intake. We assessed the percentage of SFS (%SFS) complied with by reviewing school food menus and observing food served in school canteens. We assessed pupil nutritional intake using a 24-hour dietary recall measure (Intake24) and estimated intakes of free sugar (primary outcome) and other nutrients/foods. We used adjusted multilevel models to compare pupil intakes in the SFS-mandated and SFS-non-mandated schools, and to explore the association between school SFS compliance and pupil intakes.

**Results:**

36 schools (23 not mandated and 13 mandated to comply with the SFS) and 2,273 pupils participated. The median %SFS complied with was 63.9% (interquartile range 60.0–70.0%). This was similar for SFS-non-mandated (64.5%) and SFS-mandated schools (63.3%). Compliance was highest for standards applying to lunchtime (median = 81.3%) and lowest for those applying across the whole school day (median = 41.7%). It was also lower for standards restricting high fat, sugar and energy-dense items (median = 26.1%) than for standards aiming to increase dietary variety (median = 92.3%). Pupils from SFS-mandated schools had a lower mean lunchtime intake of free sugar (g) (adjusted mean difference: -2.78g; 95% CI: -4.66g to -0.90g). There were few significant associations between %SFS complied with and pupil nutritional intake.

**Conclusions:**

English secondary schools do not fully comply with SFS legislation regardless of whether they are mandated to comply. Schools and caterers may require monitoring and support to fully comply. There is little evidence that SFS compliance is associated with better pupil nutritional intake. Food environments outside of school also need to be considered.

**Study registration:**

ISRCTN68757496 (17-10-2019).

**Supplementary Information:**

The online version contains supplementary material available at 10.1186/s12966-024-01672-w.

## Introduction

Adolescence is a major risk period for obesity development [1], with nearly a quarter of 15-year-olds living with obesity in England by 2019 [2]. Adolescent dietary intake is generally of low quality in the UK, with high consumption of free sugar (average 12.5% of daily energy intake) and sugar-sweetened beverages, and low consumption of fibre, and fruit and vegetables. Only 12% of adolescents aged 11–18 years have the recommended five fruit and vegetable portions per day [3]. The risk of obesity and poor dietary intake is variable across England, with adolescents from more socioeconomically disadvantaged families, and from some ethnic minority communities at greater risk [4, 5]. Schools are opportune settings for dietary interventions as almost all children up to 16 years attend school, consuming at least one meal during the school day. Therefore, school-based intervention and school food policies have the potential to reach children and adolescents who are at higher risk of poor diets and obesity. Interventions directly influencing school food provision include those aiming to improve the nutritional quality of school meals (e.g. standards for school meals) and interventions to provide additional healthy food (e.g. school fruit and vegetable schemes). In the European region, almost all countries have policies or guidelines for school food provision, with the aim of improving child nutrition, reducing childhood obesity and encouraging healthier dietary habits [6]. The international evidence suggests that these types of policies and interventions may have modest, short-term impacts on some aspects of the diet, such as increasing fruit and decreasing fat and sugar-sweetened beverage intake [7–10]. 

In England, legislation on nutritional school food standards (SFS) was introduced from 2006 [11] and revised in 2015 [12]. The current SFS are food-based and grouped into standards applying: only to school lunch; only to food provision outside of lunchtime; and to food provision across the whole school day. They cover six food groups (starchy foods; fruit and vegetables; milk and dairy; meat, fish, eggs, beans and other non-dairy sources of protein; foods high in fat, sugar and salt; and healthier drinks) and also address portions, variety and frequency of provision. Broadly, the SFS aim either to increase variety and balance of nutritional intake, or to restrict consumption of foods and drinks that are energy-dense and high in fat, salt and sugar [13]. State-funded schools are mandated to comply with the school food standards (SFS). However, historically, within the previous (2006) SFS legislation, a group of schools (academies and free schools established between 2010 and 2014) were exempt from having to comply with the standards, and these schools remained exempt from the 2015 legislation when it was introduced [14]. These schools were instead encouraged to voluntarily comply with the SFS [15]. There are currently no external monitoring arrangements for SFS compliance in England [16, 17]. 

Evidence for the impact of the English SFS on the nutritional intake of school pupils mainly comes from studies with children under 12 years of age. An evaluation of the change in dietary intake in children aged 4–7 years and 11–12 years following the introduction of the 2006 SFS showed improvements in overall nutritional intake in the younger, but not the older age-group [18–20]. Another English primary school-based study (children aged 4–11 years) examined the change in food provision and consumption in 136 schools after introduction of the 2006 SFS, and reported healthier food provision and increased lunchtime fruit and vegetable consumption [21]. Evidence for the impact of the SFS on the dietary intake of secondary school pupils (aged 11–16 years) is limited. A study in 80 secondary schools observed an increase in the nutritional quality of food provided, a reduction in the availability of confectionery and small improvements in lunchtime nutritional intake, following introduction of the 2006 SFS [22]. In addition, little is known about the level of compliance with the standards in English secondary schools, given that there is no external monitoring system. Therefore, in this paper we present findings from a wider study of national school food policy (the FUEL study [23]) on SFS compliance in English secondary schools, and how this is associated with the nutritional intake of secondary school pupils. Specifically, in this paper we address the following research questions: 1) In secondary schools mandated to comply with the SFS vs. schools which are exempt, are there differences in (a) compliance with the SFS, and (b) the nutritional intake of school pupils?; and 2) Is the level of compliance with the SFS associated with the nutritional intake of secondary school pupils?

## Methods

### Design and setting

We conducted a cross-sectional study in secondary schools in the Midlands, England. We recruited schools which were mandated or not mandated to comply with the SFS legislation to enable comparisons of SFS compliance and pupil nutritional intake across the two school groups.

### Sampling and recruitment

#### Schools

We identified all mainstream secondary schools classed as academies and free schools (comprising 80% of all state-funded secondary schools [24]) in 22 Local Authority areas in the Midlands, England, using Department for Education data [25]. These areas have high ethnic diversity and deprivation [26, 27]. We excluded specialist provision academies and schools that did not include the relevant year groups. We determined each school’s SFS status (mandated or not mandated) by identifying the date the school was established. We obtained routinely collected data on school characteristics: local authority area; school type; urban/rural; number of pupils; school income deprivation affecting children index (IDACI) [28]; and the percentage of pupils who were female, from minority ethnic groups, classed as having English as an additional language (EAL), classed as having special educational needs (SEN), and eligible for free school meals (FSM) [25]. We generated propensity scores by regressing school SFS-mandated or non-mandated status on these characteristics, and used them to stratify our sampling to improve comparability across the two school groups. We used propensity score quartile boundaries to generate four sampling strata and divided each into two groups based on SFS-mandated status. We invited schools in each sampling group to participate in a random order.

#### Pupils

We identified class groupings in year groups 7 (11–12 years), 9 (13–14 years) and 10 (14–15 years) which were not related to academic ability or self-selected subjects. We invited all pupils in the selected class to participate with no exclusions. We obtained passive consent from parents and written (active) assent from pupils. Recruitment of schools and participants took place between November 2019 and April 2022 (no recruitment from March 2020 and May 2021 due to the COVID-19 pandemic). Schools received £300 and pupils received £5 as a reward for participating.

#### Sample size

We estimated the required sample size using free sugar intake as the primary outcome. Based on a difference of free sugar intake of 4 g between pupils attending SFS-mandated vs. SFS non-mandated schools [29] (assuming a SD of 11, an intra-cluster correlation coefficient (ICC) of 0.1 [30] and balanced cluster sizes), we estimated that a sample of 22 schools and 990 pupils in each group (total schools = 44, total pupils = 1980, average cluster size = 45) would give over 90% power at the 5% significance level. Following COVID-19 pandemic interruptions, with the approval of the Study Steering Committee, we revised the sample size estimation based on the data already collected. We had higher cluster sizes (average pupils = 68 per school) and higher numbers of schools in the SFS non-mandated sampling groups, therefore, we estimated that a sample size of 14 schools in the SFS-mandated, and 20 schools in the SFS non-mandated groups would enable us to detect a difference of 4 g in free sugar intake with 87% power at the 5% significance level.

### Assessment of school compliance with the national SFS

For each school we collected menus for all school food outlets, covering all service periods. We also identified whether the school had in-house catering or an external school catering contractor. To capture ‘off menu’ foods and drinks available, we designed and piloted an observation tool (Additional File 1) and used it to observe all food outlets across one whole school day. We developed criteria to assess SFS compliance using the UK Statutory Instrument for The Requirements for School Food Regulations 2014 [12] and School Food Standards Practical guidance [13]. Two researchers independently assessed the menu and observation data against these criteria to make a judgement on whether schools were complying with each of the 32 SFS (further detail is provided in Additional File 2). Judgments were compared and discrepancies resolved, and a final check of SFS judgements across all schools was made to ensure consistency. We calculated the percentage of SFS (%SFS) complied with in each school overall, and then separately for the SFS applying to: school lunch; food provision outside of lunchtime; and food provision across the whole school day. Where a judgement could not be made on compliance with an individual standard, we excluded the standard from the denominator for that school. To further explore SFS compliance, we identified two types of standards (Additional File 3); those aiming to increase dietary variety (*n* = 15), and standards aiming to restrict high fat, sugar and energy-dense foods and drinks (*n* = 12). We calculated the proportion of these two SFS types that were complied with in each school. The remaining five SFS were excluded as they did not specifically relate to increasing nutritional variety or restricting high fat, sugar and energy-dense items (Additional File 3). Finally, to explore the relationship between school SFS compliance and socioeconomic deprivation, we plotted %SFS compliance against school IDACI, an index which indicates the proportion of all children aged 0–15 years living in income deprived families in the area local to the school.

### Assessment of pupil nutritional intake

#### Nutritional intake variables

In pupil participants we assessed intake of: free sugars (g; primary outcome); percentage energy intake from free sugars; energy intake (kcal); fats (g); fibre (Association of Analytical Chemists (AOAC) method; g); fruit and vegetable (F&V) portions; number of sugar-sweetened beverages (SSB); number of confectionery items (including chocolate); and number of HFSS items (defined using the Nutrient Profiling Model [31]). We assessed nutritional intakes at: school lunch, across the whole school day, and over 24 h. We also assessed free sugar intake providing > 5% energy intake; the number of eating/drinking occasions per day (excluding plain water); and consumption of five or more F&V portions per day.

#### Nutritional intake measurement

We collected nutritional intake data using Intake24; an online self-completion 24-hour recall tool that is based on the multiple pass method and has been validated for use in adolescents [32, 33]. The tool matches foods to the National Diet and Nutrition Survey (NDNS) food database, which includes over 2300 items, and is linked to the UK Composition of Foods Integrated Dataset [34]. To improve applicability of Intake24 to an ethnically diverse population we adapted it by including commonly consumed culturally diverse foods. Through piloting and consultation with members of minority ethnic communities and local nutritionists, we identified and added 63 foods (Additional File 4), obtaining nutrient composition data for these from other food composition sources [35] or by matching to existing Intake24 items.

We aimed for all participating pupils to provide nutritional intake data covering the full 24-hour period for two non-consecutive school days, however, due to school closures in the COVID-19 pandemic and pupil absences, some participants only provided data for one complete school day. Participants completed Intake24 in a timetabled session in school, with a researcher present on the first occasion and a teacher present on the second occasion. Participants entered all foods and drinks consumed, indicating portion size, the time consumed and the eating occasion (e.g. breakfast, lunch). For each eating occasion we asked participants to provide the source of the items consumed. For school lunchtime consumption we generated a ‘lunch source’ binary variable: 100% school-provided lunch vs. other (e.g. obtained from home, shop, café, takeaway, or a variety of sources). We asked participants to provide a location of consumption for each eating occasion. From these data, we generated the specified nutritional intake variables. We defined intake at school lunch as any intake marked ‘lunch’ in Intake24, and intake across the school day as any items consumed between 9.00 am and 2.00 pm, and items outside of these times that were consumed on the school premises (to account for the variation in the length of the school day across participating schools).

### Pupil sociodemographic data collection

We asked participants to complete an online survey (REDCap [36]) which included questions on date of birth, sex (male, female, other/unknown), ethnicity (using the 2011 census classification [37]), postcode data and receipt of FSM (in the UK only families on low incomes are eligible to receive FSM [38]). We combined ethnicity classifications to generate five groups (White, Asian/Asian British, Black/African/Caribbean/Black British, Mixed/Multiple, Other ethnic group/unknown). We mapped postcodes to the English Index of Multiple Deprivation (IMD) 2019 scores [28] and categorised into five groups using national quintile cut-off points.

### Analysis of pupil nutritional intake

#### Comparison of pupil nutritional intake across SFS-mandated and SFS non-mandated schools

We conducted all analyses using Stata v17. We summarised nutritional intakes for participants by SFS-mandated/non-mandated school group. Using multilevel linear or Poisson regression models we compared intakes of free sugar (g), and a further eight nutritional variables (described above) for pupils from SFS-mandated and SFS-non-mandated schools for school lunch, the whole school day, and 24 h. We compared three additional variables using multilevel logistic (> 5% energy intake from free sugar, *≥* 5 F&V portions per day) or Poisson (number of eating/drinking occasions) regression models. We initially constructed the models with random effects allowing for repeated dietary intake measures and clustering of students within year groups and schools, but during analysis we observed that the model fits between year as a random effect and year as a fixed effect were similar. We therefore simplified the models by including year group as a fixed effect. We included additional pupil-level covariates (age, sex, ethnicity, IMD group and lunch source) and school-level covariates (in-house/external school food catering, percentage of pupils eligible for FSM, and academic year of data collection (due to potential COVID-19 pandemic effects)) as fixed effects. For the primary outcome model, we initially included all school-level variables used for generating propensity scores and conducted a backward elimination process using an alpha value of 0.1. This resulted in retention of school IDACI, presence of a sixth form and religious status in the model as fixed effects. We included these additional school-level covariates in all other models. We presented estimates of regression coefficients, incidence rate ratios (IRR) and odds ratios (OR) with 95% confidence intervals (CI) and used an alpha value of 0.05 to determine statistical significance. We calculated goodness of fit statistics (Akaike Information Criterion (AIC), Bayesian Information Criterion (BIC), log likelihood) for the models.

To explore subgroup effects, we separately tested two-way interactions between SFS-mandated/non-mandated status, and lunch source, year group, and IMD quintile group in the models. We performed sensitivity analyses using imputed data for missing age and IMD information. We inputted age using average age of participants in the relevant year group at the school, and IMD using the average IMD of participants attending the school. To explore the impact of implausible dietary intake reporting we conducted further sensitivity analyses in which we excluded participants with a total 24-hour energy intake of < 400 kcal or > 4000 kcal [39]. 

#### Exploration of the association between SFS compliance and pupil nutritional intake

As we observed wide variation in SFS compliance in both school groups, we conducted analyses to explore the association between pupil nutritional intake, and the %SFS met that were (a) aiming to increase dietary variety and (b) aiming to restrict high fat, sugar and energy-dense items. We constructed multilevel models with each nutritional intake variable as the outcome in the same way as described above, including both %SFS compliance variables as explanatory variables.

## Results

### Schools and participants

**Fig. 1:**
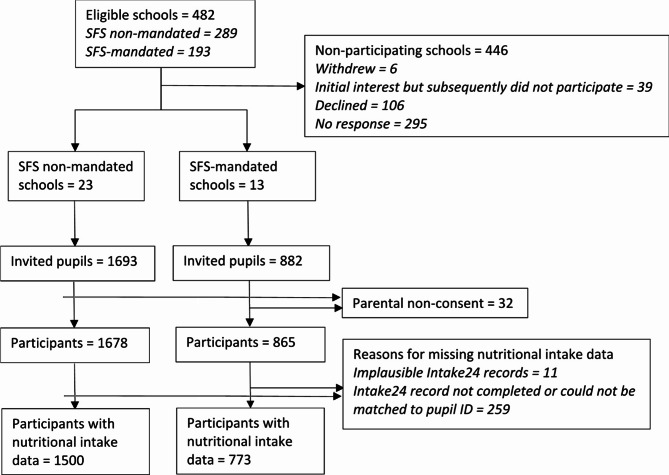
Recruitment of schools and participants

We recruited 36 schools (7.5%) from a sampling frame of 482; 23 not mandated and 13 mandated to comply with SFS legislation (Fig. 1). We invited 2,575 pupils from year groups 7, 9 and 10 to participate, and 2,543 (99%) gave their assent with passive parental consent. Of these, 2,273 (88.3% of those invited) provided nutritional intake data.

School and participant characteristics are presented in Tables 1 and 2. Compared with SFS non-mandated schools, SFS-mandated schools had a lower percentage of female, minority ethnicity pupils, and pupils who had EAL, and higher indicators of deprivation (%FSM, IDACI). There were higher proportions of male participants and participants from more deprived areas in the SFS-mandated schools than in the SFS non-mandated schools. 46% of participants contributed two 24-hour dietary recall records, and the proportion was lower in the SFS-mandated school group compared with the SFS non-mandated group (37% vs. 51%).

**Table 1:** Characteristics of participating schools

Characteristic	All schools *N* = 36 mean (SD) or *n* (%)	SFS-non-mandated schools *N* = 23 mean (SD) or *n* (%)	SFS-mandated schools *N* = 13 mean (SD) or *n* (%)
**Mean number of pupils (SD)**	1,084.36 (287.77)	1,124.26 (325.30)	1,013.77 (197.70)
**% Female**	54.95 (15.55)	57.69 (18.99)	50.09 (2.02)
**% Minority ethnicities**	29.33 (23.30)	32.40 (25.72)	23.90 (17.90)
**% EAL**	13.85 (16.28)	15.77 (18.08)	10.47 (12.43)
**% FSM**	19.77 (12.67)	18.13 (14.69)	22.68 (7.65)
**% SEN**	11.62 (4.11)	11.88 (4.44)	11.16 (3.55)
**IDACI**	0.15 (0.11)	0.13 (0.12)	0.18 (0.09)
**School type** ^**a**^			
Academy converter	23 (63.89)	19 (82.61)	4 (30.77)
Academy sponsor-led	13 (36.11)	4 (17.39)	9 (69.23)
**Presence of Sixth Form**	26 (72.22)	16 (69.57)	10 (76.92)
**Religious status**			
Faith school	3(8.33)	2 (8.70)	1 (7.69)
Secular	33 (91.67)	21 (91.30)	12 (92.31)
**Location**			
Rural	6 (16.67)	5 (21.74)	1 (7.69)
Urban	30 (83.33)	18 (78.26)	12 (92.31)
**Catering provision**			
External	21 (58.33)	13 (56.52)	8 (61.54)
In-house	15 (41.67)	10 (43.48)	5 (38.46)
**Academic year of data collection**			
2019/2020	12 (33.33)	7 (30.43)	5 (38.46)
2020/2021	4 (11.11)	3 (13.04)	1 (7.69)
2021/2022	20 (55.56)	13 (56.52)	7 (53.85)

SFS = School Food Standards; SD = Standard Deviation; % EAL = % pupils with English as an Additional Language; % FSM = % pupils eligible for Free School Meals; % SEN = % pupils with Special Educational Needs; IDACI = Income Deprivation Affecting Children Index

^a^Academy converter schools have chosen to convert to academy status; sponsor-led academy schools have been required to convert to academy status with the support of a sponsor [62]

**Table 2:** Participant characteristics

Characteristic	Total *n* = 2,273 *n* (%)	Attending SFS-non-mandated schools *n* = 1,500 *n* (%)	Attending SFS-mandated schools *n* = 773 *n* (%)
**Year group**			
7	736 (32.38)	476 (31.73)	260 (33.64)
9	796 (35.02)	535 (35.67)	261 (33.76)
10	741 (32.60)	489 (32.60)	252 (32.60)
**Age (years); mean (SD)**	13.66 (1.28)	13.66 (1.25)	13.67 (1.32)
Missing	50	31	19
**Sex**			
Female	1,269 (55.83)	878 (58.53)	391 (50.58)
Male	928 (40.83)	575 (38.33)	353 (45.67)
Other/unknown	76 (3.34)	47 (3.13)	29 (3.75)
**IMD quintile group**			
1 (highest deprivation)	543 (26.08)	310 (22.40)	233 (33.38)
2	338 (16.23)	191 (13.80)	147 (21.06)
3	413 (19.84)	261 (18.86)	152 (21.78)
4	384 (18.44)	297 (21.46)	87 (12.46)
5 (lowest deprivation)	404 (19.40)	325 (23.48)	79 (11.32)
Missing	191	116	75
**Ethnicity**			
White	1,576 (69.34)	1009 (67.27)	567 (73.35)
Asian/Asian British	359 (15.79)	269 (17.93)	90 (11.64)
Black/African/Caribbean/Black British	123 (5.41)	86 (5.73)	37 (4.79)
Mixed/Multiple	128 (5.63)	77 (5.13)	51 (6.60)
Other ethnic group/unknown	87 (3.83)	59 (3.93)	28 (3.62)
**Free School Meals**			
Yes	269 (15.35)	164 (13.64)	105 (19.09)
No	1,392 (79.45)	980 (81.53)	412 (74.91)
Pupil did not know	91 (5.19)	58 (4.83)	33 (6.00)
Missing	521	298	223
**Number of 24-hour dietary recall (Intake24) records**			
1	1,227 (53.98)	742 (49.47)	485 (62.74)
2	1,046 (46.02)	758 (50.53)	288 (37.26)

SFS = School Food Standards; SD = Standard Deviation; IMD = Index of Multiple Deprivation

### Compliance of schools with the national School Food standards

**Fig. 2:**
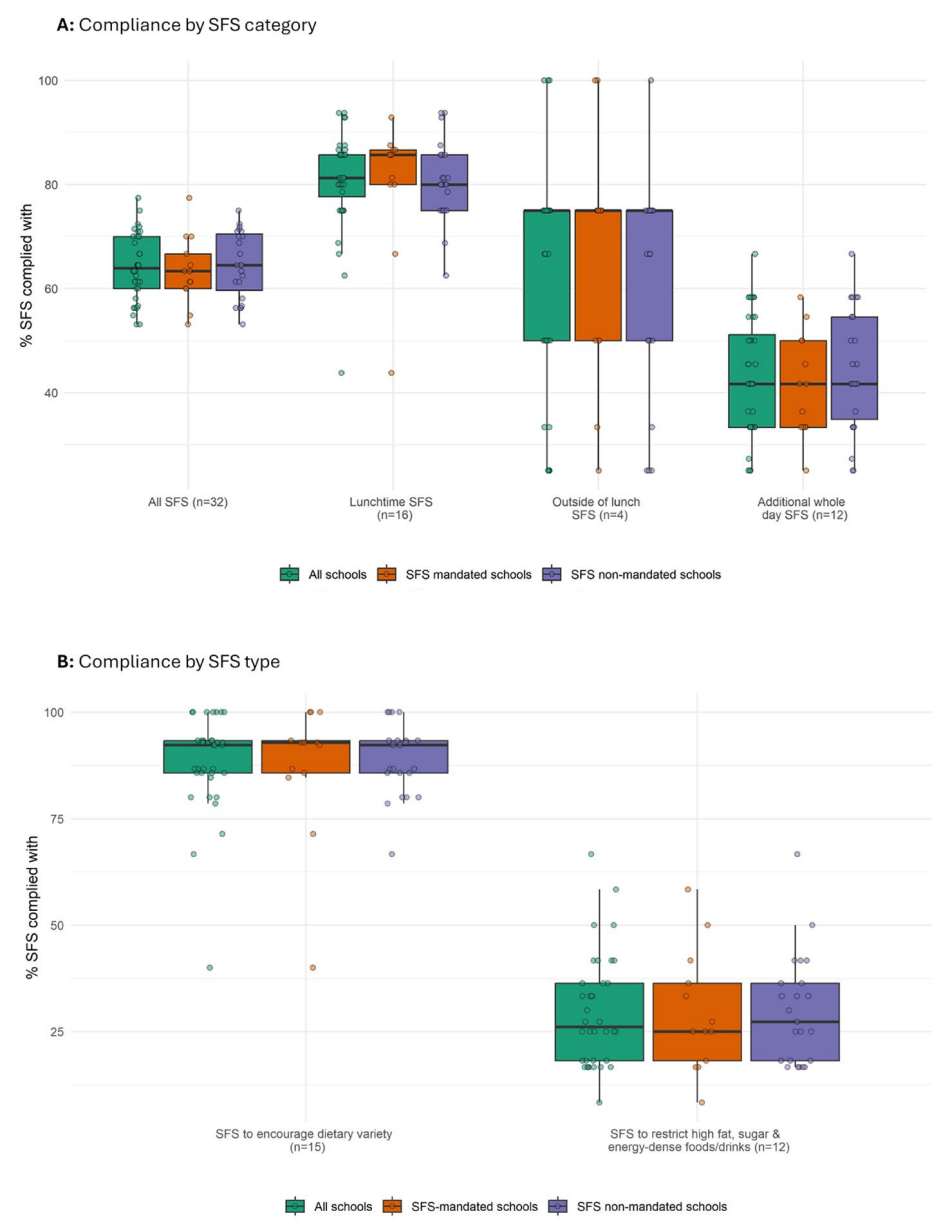
Box plots summarising percentage of School Food standards (SFS) complied with by schools

Figure 2 displays compliance of schools with the SFS overall and by SFS-mandated status. Across all schools, the median %SFS complied with was 63.9% (IQR 60.0–70.0%) with similar levels of compliance for SFS non-mandated and SFS-mandated schools. No school achieved 100% compliance with the SFS, with the percentage ranging from 53.1 to 77.4% of standards met across the 36 schools. Examining SFS categories, we observed the highest compliance with the SFS applying to school lunch, with a median %SFS compliance of 81.3% (IQR 76.8–85.7%), and the lowest compliance with the SFS applying across the whole school day (median %SFS compliance 41.7%; IQR 33.3–52.3%). On exploration of levels of compliance with our two identified types of standards, we observed a marked difference with a median %SFS compliance of 92.3% (IQR 85.7–93.3%) and 26.1% (IQR 18.2–36.4%) for SFS to encourage dietary variety and SFS to restrict high fat, sugar and energy-dense items, respectively. The median %SFS compliance for the different SFS categories and types was similar for the SFS non-mandated and SFS-mandated school groups. We did not detect any clear relationship between %SFS compliance and school IDACI (see Additional File 5). We provide detail on compliance with individual standards in Additional File 6.

### Comparison of pupil nutritional intake in SFS-mandated and SFS non-mandated schools

Table 3 displays the mean nutritional intakes of pupils in SFS non-mandated and SFS-mandated schools, and the adjusted mean differences (or IRR/OR) at lunch, across the whole school day and over 24 h. The mean intake of free sugar (g) at lunch was lower in participants from the SFS-mandated school group with an adjusted mean difference of -2.8 g (95% CI: -4.7 to -0.9 g; *p* = 0.004), but there were no significant differences in free sugar intake during the school day or over 24 h. Other significant differences in nutritional intake in the adjusted comparisons between the two school groups included a lower lunchtime energy intake (-54.97 kcal; 95% CI: -88.87 to -21.07 kcal; *p* = 0.001) and fat intake (-2.19 g; 95% CI: -3.79 to -0.58 g; *p* = 0.01) in the SFS-mandated school group, although differences in these intakes in the whole school day and 24-hour intake models were non-significant. F&V intake at lunchtime (mean difference=-0.24 portions; 95% CI: -0.36 to -0.11 portions; *p* < 0.001), across the whole school day (mean difference=-0.21 portions; 95% CI:-0.34 to -0.08 portions; *p* = 0.002) and during 24 h (mean difference=-0.35 portions; 95% CI: -0.64 to -0.06 portions; *p* = 0.02) were also significantly lower in the SFS-mandated school group.

**Table 3:** Participant nutritional intake at lunch, across the whole school day, and during 24 h: mean intakes by SFS non-mandated/SFS-mandated school status and adjusted mean differences/incidence rate ratios/odds ratios

Nutritional intake variable	Lunch intake^a^			School day intake^b^			24-hour intake		
	SFS-non-mandated *n* = 1,373 mean (SD)	SFS-mandated *n* = 715 mean (SD)	Adjusted Mean difference/IRR (95% CI); *p*-value^c^ *n* = 1,878^d^	SFS-non-mandated *n* = 1,414 mean (SD)	SFS-mandated *n* = 734 mean (SD)	Adjusted Mean difference/IRR (95% CI); *p*-value^c^ *n* = 1,934^d^	SFS-non-mandated *n* = 1,500 mean (SD)	SFS-mandated *n* = 773 mean (SD)	Adjusted Mean difference/IRR/OR (95% CI); *p*-value^c^ *n* = 2,045^d^
**Free sugar (g)**	16.89 (19.83)	15.58 (18.40)	**-2.78 (-4.66**,** -0.90); 0.004**	26.17 (29.30)	27.98 (33.81)	0.22 (-3.02, 3.47); 0.89	72.33 (63.01)	75.36 (61.88)	1.15 (-5.30, 7.61); 0.73
**% energy intake from free sugar**	12.26 (13.40)	12.03 (13.68)	-0.90 (-2.76, 0.95); 0.34	13.84 (12.82)	14.09 (12.68)	-0.31 (-2.04, 1.42); 0.72	14.77 (9.24)	15.77 (9.54)	0.77 (-0.53, 2.07); 0.25
**Energy intake (kcal)**	540.57 (352.05)	503.12 (317.90)	**-54.97 (-88.87**,** -21.07); 0.001**	714.59 (484.98)	701.27 (482.81)	-31.95 (-75.88, 11.99); 0.15	1,785.23 (880.46)	1,736.38 (876.57)	-78.71 (-175.43, 18.01); 0.11
**Fat intake (g)**	21.72 (17.16)	20.34 (15.02)	**-2.19 (-3.79**,** -0.58); 0.01**	29.01 (23.55)	28.13 (21.09)	-1.39 (-3.48, 0.69); 0.19	68.81 (40.25)	67.19 (38.83)	-2.98 (-7.10, 1.14); 0.16
**Fibre intake (g)**	4.81 (3.29)	4.57 (3.26)	-0.25 (-0.69, 0.18); 0.26	6.14 (4.24)	6.00 (4.36)	-0.17 (-0.65, 0.31); 0.48	15.81 (8.28)	14.87 (8.18)	-0.96 (-2.11, 0.19); 0.10
**F&V portions**	0.86 (1.06)	0.61 (0.84)	**-0.24 (-0.36**,** -0.11); <0.001**	1.06 (1.30)	0.83 (1.10)	**-0.21 (-0.34**,** -0.08); 0.002**	2.74 (2.21)	2.30 (2.10)	**-0.35 (-0.64**,** -0.06); 0.02**
**Number of SSB items**	0.09 (0.26)	0.12 (0.31)	1.11^e^ (0.85, 1.46); 0.45	0.14 (0.37)	0.18 (0.44)	1.16^e^ (0.85, 1.58); 0.36	0.37 (0.69)	0.48 (0.77)	1.29^e^ (1.00, 1.65); 0.05
**Number of HFSS items**	0.93 (0.92)	0.89 (0.92)	0.92^e^ (0.83, 1.01); 0.09	1.44 (1.17)	1.39 (1.22)	0.97^e^ (0.89, 1.05); 0.43	3.01 (1.89)	2.94 (1.93)	0.99^e^ (0.92, 1.06); 0.72
**Number of sugar/chocolate** **confectionery items**	0.13 (0.34)	0.13 (0.32)	0.97^e^ (0.75, 1.25); 0.81	0.23 (0.44)	0.25 (0.46)	**1.21**^**e**^**(1.01**,** 1.46); 0.04**	0.43 (0.66)	0.42 (0.61)	1.04^e^ (0.91, 1.20); 0.56
**> 5% of 24-hr energy intake from free sugar**							1,323 (88.20)^f^	693 (89.65)^f^	1.28^g^ (0.94, 1.75); 0.12
**5 or more portions F&V/24-hrs**							194 (12.93)^f^	65 (8.41)^f^	0.70^g^ (0.49, 1.02); 0.06
**Number of eating/drinking occasions/24-hrs**							4.23 (1.17)	4.14 (1.22)	0.99^e^ (0.95, 1.04); 0.81

SFS = School Food Standards; SD = Standard Deviation; IRR = Incidence Rate Ratio; OR = Odds Ratio; F&V = Fruit and Vegetable; SSB = Sugar Sweetened Beverage; HFSS = High Fat Sugar Salt

^a^Participants reporting zero calorie intake at lunch are excluded (*n* = 185)

^b^Participants reporting zero calorie intake during the school day are excluded (*n* = 125)

^c^Model covariates: sex, age, ethnicity, IMD quintile group, lunch source, school % FSM, school IDACI, school six form, school catering provision, school religious status, data collection year, year group

^d^Participants with missing covariate data are excluded from the models

^e^IRR

^f^n (%)

^g^OR

To explore whether the differences in intakes that we observed between the two school groups could be explained by the lower energy intake in the SFS-mandated school group, we re-ran the models with an additional covariate of energy intake (Additional File 7). The differences in free sugar or fat intake at lunch were no longer significant but F&V consumption remained significantly lower in the SFS-mandated school group.

Few of the interaction terms (between SFS-mandated/non-mandated status, and lunch source, year group, and IMD quintile group) included in the models, were significant (Additional File 8), indicating that there were no clear subgroup differences in the associations between school SFS-status and nutritional intake. We compared regression coefficients from the models in our main analyses with our three sets of sensitivity analyses and did not observe any substantial differences. The significant difference in the primary outcome of free sugar intake at lunch remained in all sensitivity analyses (Additional File 9).

### Compliance with SFS to encourage dietary variety and SFS to restrict high fat, sugar and energy-dense items, and associations with pupil nutritional intake

Table 4 displays the regression analyses exploring the associations between compliance with standards to encourage dietary variety and compliance with standards to restrict high fat, sugar and energy-dense items, and nutritional intake. We observed mixed associations between the %SFS to encourage dietary variety that were complied with and nutritional intake. Compliance with these standards was positively associated with the percentage of energy intake from free sugar at lunch (0.14%; 95% CI: 0.07 to 0.22%; *p* < 0.001) and across the whole school day (0.12%; 95% CI: 0.05 to 0.20%; *p* = 0.001), but inversely associated with lunchtime intake of energy (-2.14 kcal; 95% CI: -3.88 to -0.41 kcal; *p* = 0.02) and fat (-0.12 g; 95%CI: -0.19 to -0.04 g; *p* = 0.003), and with fibre both at lunchtime (-0.03 g; 95% CI: -0.05 to -0.01 g; *p* = 0.001) and across the whole school day (-0.02 g; 95% CI: -0.05 to -0.002 g; *P* = 0.03). There were no significant associations between the compliance with SFS to encourage dietary variety and 24-hour nutritional intake.

**Table 4:** Adjusted associations between %SFS to encourage dietary variety and %SFS to restrict high fat, sugar and energy-dense items complied with by schools, and pupil nutritional intakes at lunch, across the whole school day, and during 24 h

Nutritional intake variable	SFS to encourage dietary variety: %SFS complied with			SFS to restrict high fat, sugar and energy-dense items: %SFS complied with		
	Lunch intake (*n* = 1,878) Coefficient/IRR (95% CI); *p*-value	School day intake (*n* = 1,934) Coefficient/IRR (95% CI); *p*-value	24-hour intake (*n* = 2,045) Coefficient/IRR (95% CI); *p*-value	Lunch intake (*n* = 1,878) Coefficient/IRR (95% CI); *p*-value	School day intake (*n* = 1,934) Coefficient/IRR (95% CI); *p*-value	24-hour intake (*n* = 2,045) Coefficient/IRR (95% CI); *p*-value
**Free sugar (g)**	0.04 (-0.05, 0.14); 0.39	0.04 (-0.11, 0.20); 0.58	0.14 (-0.17, 0.44); 0.37	0.06 (-0.03, 0.14); 0.18	0.11 (-0.02, 0.25); 0.11	0.29 (0.03, 0.56); 0.03
**% Energy intake from free sugar**	**0.14 (0.07**,** 0.22);** **< 0.001**	**0.12 (0.05**,** 0.20);** **0.001**	0.05 (-0.01, 0.12); 0.11	0.02 (-0.05, 0.09); 0.52	0.03 (-0.03, 0.10); 0.32	0.03 (-0.03, 0.09); 0.30
**Energy intake (kcal)**	**-2.14 (-3.88**,** -0.41);** **0.02**	-1.10 (-3.28, 1.07); 0.32	-1.07 (-5.78, 3.64); 0.66	0.18 (-1.33, 1.68); 0.82	1.26 (-0.62, 3.14); 0.19	3.59 (-0.49, 7.68); 0.09
**Fat intake (g)**	**-0.12 (-0.19**,** -0.04);** **0.003**	-0.06 (-0.16, 0.04); 0.24	-0.13 (-0.34, 0.07); 0.19	-0.006 (-0.07, 0.06); 0.86	0.04 (-0.05, 0.13); 0.40	0.07 (-0.10, 0.24); 0.42
**Fibre intake (g)**	**-0.03 (-0.05**,** -0.01);** **0.001**	**-0.02 (-0.05**,** -0.002);** **0.03**	-0.01 (-0.07, 0.05); 0.68	0.004 (-0.01, 0.02); 0.61	0.01 (-0.009, 0.03); 0.30	0.03 (-0.03, 0.08); 0.34
**F&V portions**	0.0002 (-0.007, 0.007); 0.96	0.0003 (-0.007, 0.007); 0.94	-0.0006 (-0.02, 0.02); 0.94	0.0000 (-0.006, 0.006); 0.99	0.003 (-0.003, 0.009); 0.39	0.004 (-0.01, 0.02); 0.60
**Number of SSB items**	1.00^a^ (0.98, 1.01); 0.87	1.00^a^ (0.98, 1.02); 0.94	1.00^a^ (0.99, 1.01); 0.95	0.99^a^ (0.98, 1.01); 0.33	1.00^a^ (0.98, 1.01); 0.51	1.00^a^ (0.99, 1.01); 0.78
**Number of HFSS items**	1.00^a^ (0.99, 1.01); 0.96	1.00^a^ (1.00, 1.01); 0.37	1.00^a^ (1.00, 1.00); 0.82	1.00^a^ (1.00, 1.01); 0.44	1.00^a^ (1.00, 1.01); 0.09	1.00^a^ (1.00, 1.01); 0.06
**Number of sugar/chocolate** **confectionery items**	1.00^a^ (0.99, 1.02); 0.79	1.00^a^ (0.99, 1.01); 0.78	1.00^a^ (0.99, 1.01); 0.61	1.01^a^ (1.00, 1.02); 0.19	**1.01**^**a**^**(1.00**,** 1.02);** **0.02**	**1.01**^**a**^**(1.00**,** 1.01);** **0.01**

IRR = Incidence Rate Ratio; CI = Confidence Interval; F&V = Fruit and Vegetable; SSB = Sugar Sweetened Beverage; HFSS = High Fat Sugar Salt

Model covariates: sex, age, ethnicity, IMD quintile group, lunch source, school % FSM, school IDACI, school six form, school catering provision, school religious status, data collection year, year group

Participants with missing covariate data are excluded from the models

^a^IRR

Regarding compliance with SFS to restrict fat, sugar and energy-dense items and nutritional intake, we observed positive associations between %SFS compliance and 24-hour free sugar intake (0.29 g; 95% CI: 0.03 to 0.56 g; *p* = 0.03), and intake of confectionery items during the whole school day (IRR = 1.01; 95% CI: 1.00 to 1.02; *p* = 0.02) and over 24 h (IRR = 1.01; 95% CI: 1.00 to 1.01; *p* = 0.01). We did not observe these associations in the lunch intake models, and there were no significant associations with other nutrient intakes.

## Discussion

### Principal findings

No secondary school participating in this study fully complied with the current English SFS legislation, regardless of whether they were mandated to do so. On average, schools complied with 64% of the standards. Schools had the highest compliance with SFS applying to school lunchtime (median compliance 81%) and the lowest compliance with SFS that apply across the whole school day (median compliance 42%). Although there was a large variation in the level of SFS compliance overall, little difference existed between SFS-mandated and SFS-non-mandated schools. This may be because the UK government has encouraged exempt schools to follow the standards since their introduction in 2015, and it is now an expectation that all schools comply [16]. On further examination of compliance with different types of SFS, we identified that schools have higher compliance with standards encouraging dietary variety and nutritional balance than with standards restricting high fat, sugar and energy-dense foods and drinks (median compliance 92% vs. 26%). There was no clear relationship between SFS compliance and deprivation of the population the schools served.

In our adjusted comparison of nutritional intake in pupils attending SFS-mandated and SFS non-mandated schools, we detected some small but significant differences in nutritional intake, however, we cannot attribute this to SFS-status as we did not detect any meaningful differences in SFS compliance between the two school groups. It could be that differences in other characteristics of the schools explain this difference in mean nutrient intake, despite our attempts to adjust for potential school- and individual-level confounding factors in the analysis. For example, the nature and density of food vendors surrounding schools may influence nutritional intake.

We found mixed associations between level of compliance and pupil nutritional intake. While higher compliance with standards to encourage dietary variety was associated with lower energy and fat intake at school lunch, it was also associated with a higher percentage of energy intake from free sugar and a lower fibre intake during the school day. We observed few associations between compliance with standards to restrict high fat, sugar and energy-dense foods and drinks and nutritional intake, however, there were small but significant positive associations with the consumption of confectionery items across the whole school day and over 24 h, and free sugar intake over 24 h.

### Comparison with other studies

We know of no other studies that have examined secondary school compliance with the 2015 SFS legislation on the scale of this study, however our findings of incomplete compliance are consistent with a London-based study conducted in 2020. The study also reported low SFS compliance but only included a small number of secondary schools [40]. Our study provides further insights, identifying that standards restricting high fat, sugar and energy-dense items are especially problematic for secondary schools. The low compliance with these SFS is corroborated by research on the nutritional intake of secondary school pupils in the UK. Studies have reported high intakes of sweet and savoury snacks, and ultra-processed foods in secondary school pupils consuming school food [41, 42]. 

Research on SFS in primary school settings (for children age 4–11 years) shows higher compliance with the standards [29], suggesting that the ability to meet the SFS in the secondary school context is more problematic. The difference in food service arrangements between primary and secondary schools may help to explain this. Secondary schools often have more extensive food provision across the school day, and include breakfast and break time service. In contrast to primary schools, a wide range of choice is commonly a key feature of secondary school food service, with cafeteria-style provision and a variety of single food and drink items on sale, in addition to the meals advertised on school menus [43]. These ‘off menu’ items may be perceived by school caterers as a necessity to ensure the revenue for a financially viable service [44], but are typically energy-dense foods and drinks that are high in fat and sugar, and are non-compliant with the SFS [43]. 

The absence of a clear association between SFS compliance and deprivation in this study is in line with several UK and international studies, which have reported a broadly similar impact of school food policy interventions on the quality of school food provided across differing levels of deprivation [45, 46], or a neutral impact on socioeconomic inequities in dietary outcomes [47]. 

In terms of the impact of the SFS on nutritional intake of pupils in secondary schools, the evidence from two studies which explored pupil nutritional intake before and after the introduction of the 2006 SFS legislation, suggested only a marginal improvement in lunchtime nutritional intake [22] and no improvement in total dietary intake [20]. To the best of our knowledge, our study is the first to focus on the updated 2015 SFS and explore the association between the level of school compliance with the SFS and pupil nutritional intake. Our findings of little or no association between SFS compliance and pupil nutritional intake in school or overall, provide more up to date evidence that as national policy, the SFS are not achieving the desired outcomes in this age group.

The finding of the small positive association between school compliance with SFS restricting high fat, sugar and energy-dense items and the consumption of confectionery items over the school day and over 24 h warrants attention. A possible explanation is that restriction of the sale of foods that are desirable to this age group in school increases the likelihood that pupils will purchase foods from elsewhere or obtain them from home and consume them in and out of school. There is evidence from the USA to suggest that food environments surrounding schools, as well as within schools, are associated with child and adolescent obesity [48]. The lack of school policies on the foods and drinks brought into secondary schools has also been highlighted as an issue [49]. This is particularly important when considering socioeconomic disparities in adolescent diets, as there is evidence from a UK study in secondary schools to suggest that pupils attending schools serving more deprived populations are more likely to leave school to buy food from local food outlets, in pursuit of food that they deem more desirable and better value for money [50]. Areas of high deprivation are also likely to have unhealthier food environments [51, 52], which further impacts on the diets of adolescents living and attending school in these areas.

### Strengths and limitations

This study has addressed a key gap in understanding the implementation and impact of SFS policy in secondary schools. It included a large sample of schools and pupils who were representative of the English population [26, 27], and involved rigorous assessment of SFS compliance and nutritional intake data. Study limitations include issues arising from the COVID-19 pandemic. We had to pause data collection between March 2020 and May 2021 due to school closures, recommencing data collection when schools were fully open in June 2021. We collected data from four schools during the summer term of 2021 when temporary food service arrangements were still in place to address issues relating to the pandemic. However, we collected data from the remaining schools when more typical food service was in place and adjusted for year of data collection in the analyses. Overall, we had a low response rate from schools. Strategies we employed to address this included identifying key individuals to approach in school leadership teams and offering schools a report detailing their compliance with the SFS. In the absence of the pandemic interruptions, we would have been able to achieve a higher response rate with these strategies. We aimed to collect at least two 24-hour dietary recall records from participants to obtain robust data on usual nutritional intake. However, due to the pandemic interruptions, we only obtained two 24-hour records for 46% of participants. Another limitation of the nutritional intake data is the possibility of misreporting. Although we used a validated dietary intake data collection method for this age group [32], misreporting is common, especially in adolescents [53]. A further limitation of this study is that we were unable to assess all potential factors that could confound the association between adherence to the SFS and pupil nutritional intake, for example the number and nature of food outlets surrounding the schools.

### Implications

Our findings suggest that secondary schools in England do not fully comply with the SFS legislation. The unique challenges of secondary school food provision may in part explain this, but another likely contributing factor is the absence of external monitoring of SFS compliance in England [54]. Introducing robust monitoring systems may help to address the incomplete SFS compliance but alongside this, secondary schools and caterers will require support to achieve full compliance whilst maintaining a financially viable school food service. The UK government has taken some steps in this direction by introducing a pilot scheme of SFS compliance checks [55], and by providing support to schools on food procurement [56, 57]. Future policy action should expand monitoring systems and provide further support to schools, particularly focusing on the provision of ‘off menu’ items outside of the main lunch service. Another potential direction for school food policy is to review the SFS and adapt them for secondary school food contexts. The government made a commitment to update the standards in their strategy to address childhood obesity in 2016-18 [58, 59], but their focus has shifted to ensuring SFS compliance [60]. 

The lack of association between SFS compliance and healthy nutritional intake has further implications for policymakers. Whilst the food provided in schools is undoubtedly important when considering nutrition in this age group, ensuring SFS compliance or updating the SFS are unlikely to lead to substantially better dietary intake on their own. Policy action on school food needs to be undertaken in tandem with actions to address local food environments and the wider food system which have considerable influence on this age group [61]. Involvement of school-aged adolescents in developing policy relating to school food and the wider food system is critical to the success of this systemic approach, and is ultimately more likely to lead to an improvement in overall diet quality in adolescents, as well as reducing socioeconomic disparities in dietary intake. Whilst the focus of this research has been on the English secondary school context, the need for an approach that encompasses multiple food environments and the wider food system is applicable to all contexts. To facilitate this approach, future research should aim to help us better understand how home, school and external food environments interact to influence nutritional intake in adolescents. This will inform school-based and other interventions and policies that are part of a wider systems approach to improving adolescent diets.

## Conclusions

Our findings indicate that there is incomplete compliance with the national SFS legislation in secondary schools in England. Schools were less likely to comply with SFS that apply to foods and drinks served across the whole school day and outside of lunchtime, and with SFS that aim to restrict high fat, sugar and energy-dense foods and drinks, implying that these standards are problematic to adhere to in the secondary school context. To facilitate SFS compliance, external monitoring and support to schools and caterers are needed, and there may be a case for a review and update of the SFS. The level of school compliance with the SFS showed little association with pupil nutritional intake, either at lunchtime, across the school day or overall, highlighting the need for policy action to address other food environments alongside school food to achieve meaningful improvements in the diets of school-aged adolescents.

## Electronic supplementary material

Below is the link to the electronic supplementary material.

Supplementary Material 1

Additional File 1: School food outlet observation tool

Additional File 2: Process of assessment of school compliance with the school food standards (SFS)

Additional File 3: School Food Standards aiming to: (A) increase dietary variety, and (B) restrict high fat, sugar and energy-dense foods/drinks

Additional File 4: Foods commonly consumed by minority ethnic communities that were identified and included in the Intake24 dietary recall tool

Additional File 5: Relationship between the percentage of school food standards (SFS) complied with and school Income Deprivation Affecting Children Index (IDACI)

Additional File 6: Numbers and % of schools meeting each school food standard (SFS)

Additional File 7: Pupils’ nutrient and food intakes on a school day: at lunch, across the whole school day, and during 24-hours - associations with SFS-mandated/non-mandated school status, additionally adjusted for energy intake

Additional File 8: Interaction effects in the models to explore differences in pupil nutritional intakes between schools mandated or not mandated to comply with the school food standards (SFS)

Additional File 9: Models to explore differences in pupil nutritional intakes between schools mandated or not mandated to comply with the school food standards (SFS) – sensitivity analyses

## Data Availability

The datasets used during the current study are available from the corresponding author on reasonable request.

## Abbreviations

AOAC: Association of Analytical Chemists
CI: Confidence Interval
EAL: English as an Additional Language
F&V: Fruit and Vegetables
FSM: Free School Meals
g: Grams
HFSS: High Fat, Salt, Sugar
ICC: Intra-cluster Correlation Coefficient
IDACI: Income Deprivation Affecting Children Index
IMD: Index of Multiple Deprivation
IRR: Incidence Rate Ratio
IQR: Interquartile Range
kcal: Kilocalories
OR: Odds Ratio
SD: Standard Deviation
SEN: Special Educational Needs
SFS: School Food Standards
SSB: Sugar-sweetened Beverages
UK: United Kingdom
USA: United States of America
