# Correction: National school food standards in England: a cross-sectional study to explore compliance in secondary schools and impact on pupil nutritional intake

**DOI:** 10.1186/s12966-024-01696-2

**Published:** 2024-12-23

**Authors:** Miranda Pallan, Marie Murphy, Breanna Morrison, Alice Sitch, Ashley Adamson, Suzanne Bartington, Alexandra Dobell, Rhona Duff, Emma Frew, Tania Griffin, Kiya Hurley, Emma Lancashire, Louise McLeman, Sandra Passmore, Irina Pokhilenko, Maisie Rowland, Vahid Ravaghi, Suzanne Spence, Peymane Adab

**Affiliations:** 1 Department of Applied Health Sciences, Murray learning Centre, University of Birmingham, Edgbaston, Birmingham, B15 2TT UK; 2 NIHR Birmingham Biomedical Research Centre, Institute of Translational Medicine, University Hospitals Birmingham NHS Foundation Trust, University of Birmingham, Birmingham, B15 2TH UK; 3 Population Health Sciences Institute, Faculty of Medical Sciences, Newcastle University, M1.151 William Leech Building, Framlington Place, Newcastle upon Tyne, NE2 4HH UK; 4 Department for Health, University of Bath, 1 West, Claverton Down, Bath, BA2 7AY UK; 5 Services for Education, Unit 3 Holt Court, Holt Street, Birmingham, B7 4AX UK; 6 The School of Dentistry, University of Birmingham, 5 Mill Pool Way, Edgbaston, Birmingham, B5 7EG UK

**Correction to: Pallan et al**.

***International Journal of Behavioral Nutrition and Physical Activity*** **(2024) 21:123**.

10.1186/s12966-024-01672-w

Following the publication of the Original Article, the author reported a typo in the Funding section.

The grant from the National Institute for Health and Care Research (NIHR) Public Health Research Programme was erroneously written as (17/92/3); while it should have been written as **(17/92/39).**

The Original Article has been corrected.

