# Supplementary material for: National school food standards in England: a cross-sectional study to explore compliance in secondary schools and impact on pupil nutritional intake
Source: Int J Behav Nutr Phys Act. 2024 Oct 24;21:123. doi: 10.1186/s12966-024-01672-w (PMC11515374; doi:10.1186/s12966-024-01672-w)
Supplement: Supplementary file 1 — Supplementary Material 1 [file 12966_2024_1672_MOESM1_ESM.docx]

**Additional File 1: School food outlet observation tool**

**Observation details**

| School ID number |  |
| --- | --- |
| Name of food service area / dining area observed |  |
| Date of observation |  |
| Meal time observed (please circle) | Breakfast / Morning break / Lunch / After school |
| Time period |  |
| Name of observer |  |

**Please complete one form for each meal time and for each food outlet.**

**Extra forms (part G – I) will be required for additional dining areas that are separate to food outlets.**

**Please arrive 10-15 minutes before the scheduled break time**

**Key:**

| **Environment observation section** | |
| --- | --- |
|  | Food service area |
|  | Dining area |
|  | Other areas |

| **Food observation section** | |
| --- | --- |
|  | Hot food |
|  | Pre-packaged sandwiches, wraps etc. |
|  | Fruit, confectionary and snacks |
|  | Cakes and desserts |
|  | Drinks (including hot drinks) and yoghurts |
|  | Deli bar |

# Food and drinks observation **For each food item please provide as much information as possible.**

| **Hot food (includes hot food counters, soup stations)** |
| --- |
| **Main dishes and side dishes - notes on dish contents/preparation** |
|  |
|  |
|  |
|  |
|  |
|  |
|  |
|  |
|  |
|  |
|  |
|  |
|  |
|  |
|  |

| **Hot food** | | |
| --- | --- | --- |
| **Nutrient info e.g. calories, fat, sugar etc. Tick all that apply** | **Serving method Tick all that apply** | |
| - Mostly provided - Mostly not provided | - Staff - Self - Mix of staff and self - Other | |
| **Salt** | | |
| **Serving method Tick all that apply** | **Ease of access Tick all that apply** | |
| - Not available - Shakers - Sachets (state size): | - Counter top - By request - N/A | |
| **Condiments (including gravy)** | | |
| **List:** | **Serving method Tick all that apply** | **Ease of access Tick all that apply** |
|  | - Not available - Bottles - Sachets (state size): - Other: | - Counter top - By request - N/A |

| **Pre-packaged sandwiches, wraps etc.** *(Note:* *Include pre-packaged salads)* | | |
| --- | --- | --- |
| Use this space to list all sandwich and salad items. Include bread-type: | | |
| **Nutrient info e.g. calories, fat, sugar etc.** *(Note: Front of Pack refers to rapid/easy read labels e.g. Traffic light label, GDA label, calories)* **Tick all that apply** | | **Pre-packaged sandwich/wrap bread types** |
| - FOP - BOP | - Not provided - Poster/card | Estimate what proportion/number of **sandwiches** were wholegrain (brown or granary) bread: |

| **Fruit, confectionary and snacks** | |
| --- | --- |
| **Fruit - type** | **Preparation** |
|  | - Whole - Chopped - Dried/tinned |
|  | - Whole - Chopped - Dried/tinned |
|  | - Whole - Chopped - Dried/tinned |
|  | - Whole - Chopped - Dried/tinned |
|  | - Whole - Chopped - Dried/tinned |
|  | - Whole - Chopped - Dried/tinned |

| **Pre-packaged snacks and confectionary: sweets, chocolate and chocolate coated products (not baked items), cereal bars, crisps, popcorn etc.**  **Description – brand, packet size, flavour** |
| --- |
|  |
|  |
|  |
|  |
|  |
|  |
|  |
|  |
|  |

| **Cakes and desserts** *(Note: include buns, pastries, sweet and savoury biscuits, sponge puddings, pies and jellies)* | | | |
| --- | --- | --- | --- |
| Notes on dish and preparation | | Contains fruit*/veg* *(Note: include only visible F&V)* | Contains confectionary (sweets and/or chocolate)  *(Note. do not include cocoa powder)* |
|  | | - Yes - No - Couldn’t tell | - Yes - No - Couldn’t tell |
|  | | - Yes - No - Couldn’t tell | - Yes - No - Couldn’t tell |
|  | | - Yes - No - Couldn’t tell | - Yes - No - Couldn’t tell |
|  | | - Yes - No - Couldn’t tell | - Yes - No - Couldn’t tell |
|  | | - Yes - No - Couldn’t tell | - Yes - No - Couldn’t tell |
|  | | - Yes - No - Couldn’t tell | - Yes - No - Couldn’t tell |
|  | | - Yes - No - Couldn’t tell | - Yes - No - Couldn’t tell |
| Notes on dish and preparation | | Contains fruit*/veg* *(Note: include only visible F&V)* | Contains confectionary (sweets and/or chocolate)  *(Note:. do not include cocoa powder)* |
|  | | - Yes - No - Couldn’t tell | - Yes - No - Couldn’t tell |
|  | | - Yes - No - Couldn’t tell | - Yes - No - Couldn’t tell |
|  | | - Yes - No - Couldn’t tell | - Yes - No - Couldn’t tell |
| **Nutrient info e.g. calories, fat, sugar etc.** *(Note: Front of Pack refers to rapid/easy read labels e.g. Traffic light label, GDA label, calories)*  **Tick all that apply** | | **Serving method Tick all that apply** | |
| - FOP - BOP | - Not provided - Poster/card | - Staff - Self - Other | |

| **Drinks** | | | | | |
| --- | --- | --- | --- | --- | --- |
| **Milk drinks - description, including brand** | | **Container size** | | **Low fat variety** | **Ease of access** |
|  | | - 220ml or less - 221250ml | - 251ml or more - Couldn’t tell | - Yes - No - N/A | - Fridge, top - Fridge, middle - Fridge, bottom |
|  | | - 220ml or less - 221-250ml | - 251ml or more - Couldn’t tell | - Yes - No - N/A | - Fridge, top - Fridge, middle - Fridge, bottom |
|  | | - 220ml or less - 221-250ml | - 251ml or more - Couldn’t tell | - Yes - No - N/A | - Fridge, top - Fridge, middle - Fridge, bottom |
|  | | - 220ml or less - 221-250ml | - 251ml or more - Couldn’t tell | - Yes - No - N/A | - Fridge, top - Fridge, middle - Fridge, bottom |
|  | | - 220ml or less - 221-250ml | - 251ml or more - Couldn’t tell | - Yes - No - N/A | - Fridge, top - Fridge, middle - Fridge, bottom |
| **Other drinks - Bottle/can/carton - description, including brand** | | **Container size** | | **Low fat variety** | **Ease of access** |
|  | | - 150ml or less - 151-330ml | - 331ml or more - Couldn’t tell | - Yes - No - N/A | - Fridge, top - Fridge, middle - Fridge, bottom |
|  | | - 150ml or less - 151-330ml | - 331ml or more - Couldn’t tell | - Yes - No - N/A | - Fridge, top - Fridge, middle - Fridge, bottom |
|  | | - 150ml or less - 151-330ml | - 331ml or more - Couldn’t tell | - Yes - No - N/A | - Fridge, top - Fridge, middle - Fridge, bottom |
| **Other drinks - Bottle/can/carton - description, including brand** | | **Container size** | | **Low fat variety** | **Ease of access** |
|  | | - 150ml or less - 151-330ml | - 331ml or more - Couldn’t tell | - Yes - No - N/A | - Fridge, top - Fridge, middle - Fridge, bottom |
|  | | - 150ml or less - 151-330ml | - 331ml or more - Couldn’t tell | - Yes - No - N/A | - Fridge, top - Fridge, middle - Fridge, bottom |
|  | | - 150ml or less - 151-330ml | - 331ml or more - Couldn’t tell | - Yes - No - N/A | - Fridge, top - Fridge, middle - Fridge, bottom |
|  | | - 150ml or less - 151-330ml | - 331ml or more - Couldn’t tell | - Yes - No - N/A | - Fridge, top - Fridge, middle - Fridge, bottom |
|  | | - 150ml or less - 151-330ml | - 331ml or more - Couldn’t tell | - Yes - No - N/A | - Fridge, top - Fridge, middle - Fridge, bottom |
|  | | - 150ml or less - 151-330ml | - 331ml or more - Couldn’t tell | - Yes - No - N/A | - Fridge, top - Fridge, middle - Fridge, bottom |
|  | | - 150ml or less - 151-330ml | - 331ml or more - Couldn’t tell | - Yes - No - N/A | - Fridge, top - Fridge, middle - Fridge, bottom |
| **Nutrient info e.g. calories, sugar, fat etc.** *(Note: Front of Pack refers to rapid/easy read labels e.g. Traffic light label, GDA label, calories)*  **Tick all that apply** | | | | | |
| - FOP - BOP | - Not provided - Poster/card | | | | |

| **Hot drinks** *(Note: describe any other hot drinks)*  **Tick all that apply** | | **Sugar/sweeteners** *(Note: describe any other sweet ‘extras’ e.g. syrup)*  **Tick all that apply** | | | |
| --- | --- | --- | --- | --- | --- |
| - Tea - Coffee | - Hot chocolate - None | - Not available - Shakers | - Sachets - N/A | Ease of access:   - Counter top - By request | - N/A |
| **List the different milk options for hot drinks (full fat, soya etc., including brands)** | | **If dairy alternative, calcium enriched?** | | | |
|  | | - Yes - No | | - Not applicable - Couldn’t tell | |
|  | | - Yes - No | | - Not applicable - Couldn’t tell | |
|  | | - Yes - No | | - Not applicable - Couldn’t tell | |

| **Yoghurts** | | | |
| --- | --- | --- | --- |
| **Description – brand, packet size** | | **Fat content** | |
|  | | - Full | - Reduced |
|  | | - Full | - Reduced |
|  | | - Full | - Reduced |
| **Nutrient info e.g. calories, fat, sugar etc.** *(Note: Front of Pack refers to rapid/easy read labels e.g. Traffic light label, GDA label, calories)*  **Tick all that apply** | | | |
| - FOP | - BOP | - Not provided | - Poster/card |

| **Deli bar: sandwiches, wraps, salads, baked potatoes etc.** (*Note:* *Deli bar describes a select-your-own fillings/toppings service section)* | | |
| --- | --- | --- |
| **Base options** | | |
| Bread/baguettes/rolls/wraps/paninis:   - White - Wholegrain / granary | - 50/50 - Other: | Baked potatoes:   - Yes - No |
| **Staff served fillings -** How many of the following were there? | | **Self-served fillings -** How many of the following were there? |
| Use this space to list fillings. Include details on whether foods were wholegrain, coated / breaded etc. | | **OR** Use this space to list fillings: |
| **Deli bar: sandwiches, wraps, salads, baked potatoes etc.** (*Note:* *Deli bar describes a select-your-own fillings/toppings service section)* | | |
| **Salad dressings (if available)** | | **Serving method Tick all that apply** |
| List: | | - Staff - Self - Other |
| Was bread available as a standalone item?  If yes, please describe the options (including any wholegrain/granary items):  Did any of these have added fat or oil *e.g. butter, garlic bread*?   - Yes - No | | Was nutrient info provided for deli items? **Tick all that apply**   - Mostly provided - Mostly not provided |

1. Food service area: **Environment**
2. How many food service areas / lines and till points were there?

*(It is a separate counter/line/till if it does any of the following: repeats what is on offer on another counter, is physically separate, or has its own line)*

|  | Total | No. in use |
| --- | --- | --- |
| 1. Hot food counters *(exclude soup stations)* |  |  |
| 1. Soup stations |  |  |
| 1. Deli bar e.g. freshly made salad, sandwich, baked potato bars/counters |  |  |
| 1. Grab-and-go food counters/fridges e.g. pre-packaged sandwiches, snacks etc. |  |  |
| 1. Fridges containing drinks |  |  |
| 1. Drinking water stations e.g. fountains, taps or jugs   *(count the station, not the number of taps/jugs)* |  |  |
| 1. Till points |  |  |

1. To what extent do you agree or disagree with the following statements?

|  | Strongly agree | Agree | Neither agree nor disagree | Disagree | Strongly disagree | Couldn’t observe |
| --- | --- | --- | --- | --- | --- | --- |
| 1. The food service areas were clean & tidy |  |  |  |  |  |  |
| 1. The food service areas were attractive |  |  |  |  |  |  |
| 1. Pupils appeared well behaved |  |  |  |  |  |  |
| 1. There was a clear traffic pattern |  |  |  |  |  |  |
| 1. The food service areas were congested |  |  |  |  |  |  |
| 1. Drinking water was readily available (bottled or free) |  |  |  |  |  |  |

1. Use this space to make any notes / observations
2. Food service area: **Service**
3. Please complete the following table

|  | Response |
| --- | --- |
| 1. Lunch service was staggered by year group | Yes □  No □  Don’t know □  N/A □ |
| 1. Staff used the same service points as pupils | Yes □  No □ |
| 1. There were obstructions that affected student movement | No obstructions □  Some obstructions □  Many obstructions □ |
| 1. How were main meals served?   (Tick all that apply) | Served by staff □  Self-serve □  Family style (at table) □ |
| 1. How were main meals dished? (Tick all that apply) | On divided trays □  On plates □  Takeaway packaging □ |

1. Approximately, how long did a typical pupil spend waiting in each service area to be served and pay?

|  | Description of service area e.g. hot/cold/salad etc. | Less than 5 mins | 5-15 mins | 16-30 mins | Over 30 mins |
| --- | --- | --- | --- | --- | --- |
| 1 |  |  |  |  |  |
| 2 |  |  |  |  |  |
| 3 |  |  |  |  |  |
| 4 |  |  |  |  |  |
| 5 |  |  |  |  |  |
| 6 |  |  |  |  |  |
| 7 |  |  |  |  |  |
| 8 |  |  |  |  |  |
| 9 |  |  |  |  |  |
| 10 |  |  |  |  |  |

1. Please detail any additional strategies used to reduce queuing times

Please detail any additional strategies used to reduce queuing times

1. Use this space for extra notes on the service
2. Food service area: **Catering staff**
3. How many catering staff did you see? *(include till staff and supervisors)* ________
4. To what extent do you agree or disagree with the following statements?

|  | Strongly agree | Agree | Neither agree nor disagree | Disagree | Strongly disagree | Couldn’t observe |
| --- | --- | --- | --- | --- | --- | --- |
| 1. Catering staff / midday supervisors engaged positively with pupils |  |  |  |  |  |  |
| 1. Catering staff encouraged pupils to experiment with new foods e.g. verbal prompts |  |  |  |  |  |  |
| 1. Catering staff encouraged pupils to eat healthily e.g. verbal prompts |  |  |  |  |  |  |
| 1. Catering staff / midday supervisors appeared happy e.g. have a positive facial expression / demeanour |  |  |  |  |  |  |

1. Did catering staff put vegetables on pupil's plates as default?

- Yes
- No
- Couldn’t tell
- No vegetables were served

1. Use this space for extra notes on catering staff
2. Food service area: **Presentation of food**
3. How would you describe the attractiveness of the **food**?

- Great: Fresh, colourful, creatively and cleanly presented
- Good: Most items fresh, colourful, cleanly presented
- Fair: Some items fresh, colourful, cleanly presented
- Poor: Not fresh, bland colours, unattractive presentation

1. Please complete the following table

|  | Response (Tick all that apply) |
| --- | --- |
| 1. Where was fruit available to purchase? | At the till □  At the beginning of the line/counter □  At another place in the canteen □  N/A □ |
| 1. Describe the accessibility/positioning of fruit to pupils | Easily accessible □  Out of reach □  On request only □  N/A □ |
| 1. Where were confectionary and high fat/salt/sugar foods and drinks available to purchase? | At the till □  At the beginning of the line/counter □  At another place in the canteen □  N/A □ |
| 1. Describe the accessibility/positioning of confectionary and high fat/salt/sugar foods and drinks to pupils | Easily accessible □  Out of reach □  On request only □  N/A □ |
| 1. Describe the accessibility/positioning of plain bottled water to pupils | Easily accessible □  Out of reach □  On request only □  N/A □ |
| 1. Describe the accessibility/positioning of yoghurt items / non-dairy alternatives to pupils | Easily accessible □  Out of reach □  On request only □  N/A □ |

1. Use this space for extra notes on the presentation of food
2. Food service area: **Food on offer versus menu (Lunch Only)**
3. Was a daily menu for main meals/dishes available to view in the canteen?

- Yes
- No

1. To what extent do you agree or disagree that today’s menu was clear and visible?

- Strongly agree
- Agree
- Neither agree nor disagree
- Disagree
- Strongly disagree
- Not applicable (no menu was available)

1. Were **all main meal dishes on the menu** available to buy throughout the service?

- Yes – all dishes on the menu were available to buy
- No – some dishes from the menu were not available / ran out during service
- No – the wrong menu was displayed
- Couldn’t tell
- Not applicable (no menu was available)

1. If no, please specify which dishes from the menu were not served / available

**Consider the main meal dishes available**

1. Were there any **extra** main meal dishes on offer/available that weren’t on the menu?

- Yes
- No
- Not applicable

1. If yes, please provide a list of extra hot, main meal dishes offered
2. Food service area: **Information and promotions**
3. Please respond to the following:

|  | Response (Tick all that apply) |
| --- | --- |
| 1. Were the names of dishes labelled at the point of choice? | Yes □  No □  Sometimes □ |
| 1. Was allergen information given at the point of choice? | Yes □  No □  Sometimes □ |
| 1. Were there signs/posters promoting healthy eating messages? | Yes □ If yes, how many? ____  No □ |
| 1. Was there a visible ‘set menu’ meal deal?   *(for multiple meal deals, select all that apply)* | Yes, inc. a vegetable/fruit □  Yes, did not inc. a vegetable/fruit □  No □ |
| 1. Was there a loyalty card / points/rewards scheme? | Yes □  No □ |
| 1. Was there another food/drink promotion offered e.g. fruit of the day? | Yes □  No □ |

1. If signs/posters promoting healthy eating messages were observed what messages were they promoting?
2. Use this space for extra notes on information and promotions e.g. description of meal deals

G) Dining area: **Environment**

1. Please rate the following:

|  | Great | Good | Fair | Poor |
| --- | --- | --- | --- | --- |
| 1. Noise levels | Very quiet  □ | Soft voices  □ | Loud talking  □ | Shouting/screaming  □ |
| 1. Condition of dining area |  | Well kempt, no repair needed  □ | Some damage, some repair needed □ | Unkempt, many repairs needed  □ |
| 1. Attractiveness of dining area | Good physical condition, bright  □ | | Poor physical condition, dark  □ | |
| 1. Condition of furniture |  | No damage, no repair needed  □ | Some damage, some repair needed □ | Damaged, many repairs needed  □ |
| 1. Attractiveness of furniture | Good physical condition, bright  □ | | Poor physical condition, dark  □ | |
| 1. Cleanliness | Clean  □ | Almost clean  □ | Satisfactory  □ | Dirty  □ |
| 1. Smell | No smell or pleasant smell  □ | Noticeable but not unpleasant smell  □ | Slightly unpleasant smell  □ | Strong, unpleasant smell  □ |

1. To what extent do you agree or disagree with the following statements?

|  | Strongly agree | Agree | Neither agree nor disagree | Disagree | Strongly disagree | Couldn’t observe |
| --- | --- | --- | --- | --- | --- | --- |
| 1. The dining room is a happy environment |  |  |  |  |  |  |
| 1. The dining room is a calm environment |  |  |  |  |  |  |
| 1. Drinking water was readily available |  |  |  |  |  |  |
| 1. There was enough seating for all pupils to sit down to eat |  |  |  |  |  |  |

1. Use this space for extra notes on the dining environment

1. How many of the following did you observe in the dining area?

|  | Number |
| --- | --- |
| 1. Vending machines |  |
| 1. Tuck shops |  |
| 1. Drinking water stations e.g. fountains, taps or jugs   *(count the station, not the number of taps/jugs)*  Don’t count those you have already counted in another form |  |

1. If there are vending machines or tuck shops please list the types of items sold

| **Drinks** | **Other foods/snacks** |
| --- | --- |
|  |  |
|  |  |
|  |  |
|  |  |
|  |  |
|  |  |
|  |  |
|  |  |
|  |  |
|  |  |
|  |  |
|  |  |
|  |  |
|  |  |
|  |  |
|  |  |
|  |  |
|  |  |
|  |  |

1. Were bins positioned away from tables? E.g. around the outside of the room

- Yes
- No
- Some bins were positioned away from tables

1. Provide a brief description of the general décor and condition of the dining area e.g. lighting, colours, types of furniture & seating, posters/TV screens, modern/outdated

H) Dining area: Information

1. Were there signs/posters promoting healthy eating messages?

- Yes If yes, how many? ____________
- No

1. If signs/posters promoting healthy eating messages were observed what messages were they promoting?
2. Use this space for extra notes on information provided in the dining area
3. Dining area: **Staff and pupil behaviour**
4. Complete the following table

|  | Response |
| --- | --- |
| 1. Teachers/staff were eating in the dining room | Yes □  No □ |
| 1. Staff were seen monitoring/observing the dining area | Yes □  No □ |
| 1. Staff were seen monitoring packed lunches specifically | Yes □  No □  Couldn’t tell □  N/A □ |
| 1. Pupils with school lunches and packed lunches were eating together in the same room | Yes □  No □  N/A □ |
| 1. Pupils appeared well behaved *(consider shoving, pushing, shouting, fighting, bullying, compliance with staff requests, politeness/manners etc.)* | Strongly agree □  Agree □  Neither agree nor disagree □  Disagree □  Strongly disagree □ |

1. Use this space for extra notes on staff and pupil behaviour
2. **Other areas** for buying/consuming foods and drinks around the school *(Note: complete only one per school)*
3. Did you observe any vending machines outside of the dining areas?

- Yes
- No
- Couldn’t observe

1. Did you observe any tuck shops outside of the dining areas?

- Yes
- No
- Couldn’t observe

1. If there were vending machines or tuck shops please list the types of items sold

| **Drinks** | **Other foods/snacks** |
| --- | --- |
|  |  |
|  |  |
|  |  |
|  |  |
|  |  |
|  |  |
|  |  |
|  |  |
|  |  |
|  |  |
|  |  |
|  |  |
|  |  |
|  |  |
|  |  |
|  |  |
|  |  |

1. To what extent do you agree or disagree that drinking water appeared to be readily available to pupils around the school?

- Strongly agree
- Agree
- Neither agree nor disagree
- Disagree
- Strongly disagree
- Unable to observe

5. Did you observe any signs/posters promoting healthy eating messages outside of the dining areas?

- Yes
- No
- Couldn’t observe

6. If signs/posters promoting healthy eating messages were observed what messages were they promoting?

7. Please list the additional areas you saw pupils eating e.g. Common rooms, class rooms, outdoors etc.

8. Please state whether you saw any additional food service / dining / food preparation areas that were not listed in your schedule

9. Use this space for extra notes on the eating and drinking environment around the school

NOTES PAGE

Use this space to write down any queries you have for catering staff or FUEL study staff
