# Supplementary material for: National school food standards in England: a cross-sectional study to explore compliance in secondary schools and impact on pupil nutritional intake
Source: Int J Behav Nutr Phys Act. 2024 Oct 24;21:123. doi: 10.1186/s12966-024-01672-w (PMC11515374; doi:10.1186/s12966-024-01672-w)
Supplement: Supplementary file 2 — Additional File 1: School food outlet observation tool [file 12966_2024_1672_MOESM2_ESM.docx]

**Additional File 2: Process of assessment of school compliance with the school food standards (SFS)**

Table A. Interpretation of SFS and definitions of food categories applied by the FUEL study team

| **Standards** | **Additional items and clarifications included throughout data collection** |
| --- | --- |
| **SCHEDULE 2 SCHOOL LUNCH REQUIREMENTS [2]/ The standards for school lunches [1]** |  |
| One or more portions of food from sub-group A1^a^ must be provided every day [2] / One or more portions of food from this group every day [1] | This includes all breads, potatoes, rice, couscous, bulgur wheat, semolina, tapioca, maize, cornmeal, noodles and pasta, oats, millet, barley, buckwheat, rye, spelt, plantain, yam, sweet potato, and cassava. Include starchy foods where fat or oil has been added before or during the cooking process: include roast or sautéed potatoes, chips, potato wedges, pre-prepared potato products, fried rice, bread, or noodles, hash browns, garlic bread, Yorkshire pudding, chapattis and naan made with fat, pancakes and waffles cooked in oil [1]. |
| A type of bread from sub-group A2^b^ must be available every day [2] / Bread - with no added fat or oil - must be available every day [1] | All types of plain bread with no added fat or oil, including brown, wholemeal, granary, white, mixtures of white and wholemeal, pitta, rolls, chapattis, naan, ciabatta, and herb bread [1]. |
| Three or more different foods from sub-group A1^a^ must be provided every week [2] / Three or more different starchy foods each week, but this requirement will not be met if the different foods are provided in a single portion [1] | The FUEL study has grouped foods based on the types of starchy foods listed in the school food standards [1] i.e. a) All types of bread b) pasta & noodles c) rice d) potatoes & sweet potatoes e) other starchy roots e.g. yams, plantain, cocoyam and cassava f) other grains e.g. millet, couscous, bulgur wheat, maize (polenta) and cornmeal. |
| One or more of the portions of food from sub-group A1^a^ that is provided every week must be wholegrain [2] / One or more wholegrain varieties of starchy food each week [1] | Starchy wholegrains include wholemeal and granary flours, wholemeal and granary breads and bread products, wholewheat pasta, brown rice and oats. [1] *As suggested by the school food standards, the FUEL team also include higher-fibre white bread, half/half wholegrain and white mixes such as 50/50 mix of brown and white rice, 50/50 whole wheat and white pasta [1].* |
| One or more portions of food from sub-group B2^c^ (vegetables) must be provided every day [2] / One or more portions of vegetables or salad as an accompaniment every day [1] | This includes vegetables in all forms, including fresh, frozen, canned, dried and juiced, as well as pulses such as beans and lentils [1]. *The FUEL study has included coleslaw and vegetable soup within this definition but have excluded vegetables within composite dishes* [3], *pizza or sandwiches as these are not considered to be an accompaniment. Included pre-packaged salads.* |
| One or more portions of food from sub-group B1^d^ (fruit) must be provided every day [2] / One or more portions of fruit every day [1] | Fruits in all forms, including fresh, frozen, canned, dried and juiced. Fruits can be provided within other dishes - for example, fruit-based desserts such as crumbles. *The FUEL team have included 100% fruit juice/smoothies in this definition. Excluded jelly squeeze or jelly without fruit pieces as unlikely to be a portion.* |
| A fruit-based dessert with a content of at least 50% fruit measured by volume of raw ingredients must be provided two or more times every week [2]/ A dessert containing at least 50% fruit two or more times each week [1] | *The FUEL team have included the following as a fruit-based dessert: fruit crumble, fruit pie, fruit sponge, fruit cobbler, fruit jelly with fruit pieces.* Fruit used as decoration or jam added to a dessert does not count towards this standard [1]. *The FUEL team have not sought recipes from catering teams in order to assess this standard. Where it is unclear if a dessert contains at least 50% fruit based on its description, the research team searched the internet and reviewed a standard recipe (e.g. BBC Good Food) for the dessert description or a similar item to make a judgement.* |
| Three or more different foods from sub-group B1^d^ (fruit) must be provided every week, but this requirement will not be met if the different foods are provided in a single portion [2]/ At least three different fruits each week [1] | Fruits in all forms, including fresh, frozen, canned, dried and juiced [1]. Fruits can be provided within other dishes - for example, fruit-based desserts such as crumbles [1]. *The FUEL team have included 100% fruit juice/smoothies in this definition.* |
| At least three different foods from sub-group B2^c^ (vegetables) must be provided every week, but this requirement will not be met if the different foods are provided in a single portion [2] / At least three different vegetables each week [1] | This includes vegetables in all forms, including fresh, frozen, canned, dried and juiced, as well as pulses such as beans and lentils [1*]. The FUEL study has included coleslaw and vegetable soup within this definition. The FUEL team allows vegetables served in composite dishes to count towards this standard. The FUEL team have excluded vegetables within sandwiches, pizza and starch-based salads (e.g. pasta salad) as they are considered unlikely to be the equivalent of a portion.* |
| A portion of food from group C^e^ must be provided every day [2]/ A portion from this food group every day [1] | This includes fresh and frozen meat, poultry, fresh, frozen and canned fish, shellfish, eggs, meat alternatives (including products such as soya and Quorn™), tofu, pulses such as beans (cannellini, kidney, pinto, borlotti, haricot, butter, but not green beans), chickpeas, lentils (red, green, brown and puy) and nuts [1]. The school food standards incorporate meat soups; meat products; and vegetarian sausages, burgers and nut cutlets in this definition [1] |
| A portion of meat or poultry must be provided on three or more days every week [2]/ A portion of meat or poultry on three or more days each week [1] | The FUEL team have included the following in this definition: roast red meat, roast poultry, red meat or poultry in dishes such as casserole, stew, pie, or curry; meat soups; meat products [1] |
| Oily fish must be provided once or more every three weeks [2]/ Oily fish once or more every three weeks [1] | Oily fish includes fresh, tinned or frozen salmon, sardines, pilchards, mackerel, herring, and fresh or frozen tuna, as well as anchovies and trout [1, 2]. Tuna only counts as an oily fish when it is fresh or frozen (not canned) [2]. *The FUEL team have made an assumption that battered / breaded fish is not oily unless otherwise stated in the menu.* |
| A portion of non-dairy sources of protein must be provided on three or more days every week [2] / For vegetarians, a portion of non-dairy protein on three or more days each week [1] | This standard incorporates meat alternatives made from soya beans (such as soya mince and tofu) and Quorn™ (in dishes such as vegetarian casserole, stew, pie, or curry); pulses such as beans (cannellini, kidney, pinto, borlotti, haricot (including baked beans), butter, but not green beans), chickpeas, lentils (red, green, brown and puy); vegetarian sausages, burgers, nut cutlets; egg served in a salad, baked potato or sandwich [1]. *The FUEL team have excluded hummus from this definition as it considered unlikely to be the equivalent of a portion. Veggie chilli etc. was assumed to have Quorn mince or beans* |
| A portion of food in sub-group D2^f^ must be provided every day / A portion of food from this food group every day | Items made from milk but not milk itself. This includes cheese, yoghurt (including plain or fruit, frozen and drinking yoghurt), or fromage frais, milk based sauces, custard (made with milk), puddings made from milk and milk-based sauces. *Yoghurt dressing should not be counted as unlikely to be a full portion. The FUEL team have included cheese added to salads, baked potatoes, sandwiches, crackers and pizza. Yoghurt dressing should not be counted as unlikely to be a full portion. Cream or mousse can be included in this definition.* |
| Savoury crackers or breadsticks which are served with food groups B or D may be provided as part of a school lunch [2] / Savoury crackers or breadsticks can be served at lunch with fruit or vegetables or dairy food [1] | Group B and D refers to fruit and vegetables and milk and dairy foods [2]. |
| Desserts, cakes and biscuits are allowed at lunchtime. They must not contain any confectionery. [1] | This standard prohibits any product containing or wholly or partially coated with chocolate and any chocolate-flavoured substance (Excluding cocoa powder used in cakes, biscuits and puddings) [1]. Examples include chocolate and chocolate products (chocolate bars, buttons, flakes, eggs, chocolate coated bars; chocolate coated biscuits; Sweets; Chewy and crunchy cereal bars; Choc ices and chocolate coated ice cream; Processed fruit bars; Chocolate sprinkles, hundreds and thousands and chocolate vermicelli [3]. *The FUEL team include the following in this definition: Cakes decorated with sprinkles, sweets or chocolate; Cookies or muffins with chocolate chips; Cakes or biscuits with chocolate frosting or coating e.g. bourbons, Pain au chocolat; Chocolate sauce e.g. with waffles; Yoghurts with chocolate balls, chocolate flakes etc. Do not include: Cake or biscuits with non-chocolate icing or frosting; Cake or biscuits with jam; Jam, marmalade, syrup or honey served as condiments* |
| **SCHEDULE 3 REQUIREMENTS FOR FOOD PROVIDED AS PART OF A SCHOOL LUNCH OR OTHERWISE [2] / Applies across the whole school day [1]** |  |
| Food from sub-group A1^a^ which is cooked in fat or oil must not be provided on more than two days each week [2] / Starchy food cooked in fat or oil no more than two days each week [1] | Includes roast or sautéed potatoes, chips, potato wedges, pre-prepared potato products, fried rice, bread, or noodles, hash browns, garlic bread, Yorkshire pudding, chapattis and naan made with fat, pancakes and waffles cooked in oil [1]. *The FUEL team have not included batter used as a coating unless it is coating a starchy item.* Where fat or oil is only added to a starchy food after the cooking process is complete (e.g. the addition of butter or fat spread to mashed or jacket potatoes), the dish would not be classed as a starchy food cooked in fat or oil, and the provision would not be restricted [3]. *The FUEL team excludes pre-packaged waffles / pancakes from this definition unless it is clear from packaging that they have been cooked in fat prior to packaging. Included poppadoms.* |
| Meat products may not be provided more than twice each week in secondary schools [2] / A meat or poultry product (manufactured or homemade and meeting the legal requirements) no more than twice each week in secondary schools [1] | Incorporates the following: sausages made from beef, lamb or pork; Burgers; Scotch pies, bridies, sausage rolls, Cornish pasty, encased meat pastry pies, cold pork pie (e.g. Melton Mowbray); Breaded or battered shaped chicken and turkey products, e.g. nuggets, goujons, burgers [1]. Bacon is not considered a meat product within the SFS [3]. The FUEL team h*ave not included chicken / turkey burgers in this definition unless it is specified that they are coated in breadcrumbs. Have not included chicken tikka, BBQ chicken, BBQ pork, pulled pork etc.* |
| No more than two portions of food that has been deep-fried, batter-coated or breadcrumb coated may be provided each week [2] / No more than two portions of food that has been deep-fried, batter-coated or breadcrumb coated each week [1] | Incorporates foods deep fried or flash fried in the kitchen or in the manufacturing process, such as chips (including oven chips), potato waffles, hash browns, samosas, plantain chips, spring rolls, doughnuts, pakora and bhajis [1]. Batter-coated and breadcrumb-coated foods includes any bought-in or homemade products such as chicken nuggets, fish fingers, battered onion rings and tempura [1]. *The FUEL team does not include roast potatoes, potato wedges, Yorkshire pudding, sweet waffles or pancakes in this definition. Include curly fries, poppadoms, falafel.* |
| No more than two portions of food which includes pastry may be provided each week [2] / No more than two portions of food which include pastry each week [1] | Includes shortcrust, flaky, filo, choux and puff, used in quiches, meat pies, fruit pies, fruit tarts, sausage rolls, pasties, samosa [1]. |
| (1) No snacks may be provided, except for nuts, seeds, vegetables and fruit with no added salt, sugar or fat [2]/ No snacks, except nuts, seeds, vegetables and fruit with no added salt, sugar or fat [1] | Snacks means pre-packaged items other than confectionery, sandwiches, cakes or biscuits which are ready to eat without further preparation and which consist of or include as a basic ingredient potato, cereals, soya, nuts, seeds, fruit or vegetables [2]. *The FUEL team include the following within this definition: crisps / nachos; salted or sweet nuts; salted or sweet popcorn. Plain popcorn is permitted* [3]. *Exclude confectionery, sandwiches, cakes or biscuits, desserts waffles, pancakes, crackers.* |
| (2) Where dried fruit is provided it must have no more than 0.5% vegetable oil as a glazing agent [2] | Where available, the research team refer to the label. If dried fruit is not sold, this is listed as N/A |
| Confectionery must not be provided [2]/ No confectionery, chocolate or chocolate coated products [1] | Confectionery includes chewing gum, cereal bars, processed fruit bars, non-chocolate confectionery (whether or not containing sugar), chocolate in any form (except hot chocolate), any product containing or wholly or partially coated with chocolate and any chocolate-flavoured substance, but excludes cocoa powder used in cakes, biscuits and puddings or in a drink listed in group E of Schedule 1 [2]. Examples include chocolate and chocolate products (chocolate bars, buttons, flakes, eggs, chocolate coated bars; chocolate coated biscuits; Sweets; Chewy and crunchy cereal bars; Choc ices and chocolate coated ice cream; Processed fruit bars; Chocolate sprinkles, hundreds and thousands and chocolate vermicelli [3]. *The FUEL team include the following in this definition: Cakes decorated with sprinkles, sweets or chocolate; Cookies or muffins with chocolate chips; Cakes or biscuits with chocolate frosting or coating e.g. bourbons, Pain au chocolat; Chocolate sauce e.g. with waffles; Yoghurts with chocolate balls, chocolate flakes etc. Do not include: Cake or biscuits with non-chocolate icing or frosting; Cake or biscuits with jam; Jam, marmalade, syrup or honey served as condiments* |
| Salt must not be available to add to food after the cooking process is complete [2]/ Salt must not be available to add to food after it has been cooked [1] | The research team identify any salt shakers or salt sachets available to pupils |
| Condiments may only be available in sachets or individual portions of no more than 10 grams or one teaspoonful [2]/ Any condiments must be limited to sachets or portions of no more than 10 grams or one teaspoonful [1] | Includes ketchup, mayonnaise, salad cream, brown sauce, chutney [1]. |
| The only drinks that may be provided are those in group E (see next column), except that whole milk may be provided for pupils up to the end of the school year in which they attain the age of five [2]. | E1: Plain drinks:   - Plain water (still or carbonated). - Lower fat milk or lactose reduced milk. - Fruit juice or vegetable juice of no more than 150mls per portion. - Plain soya, rice or oat drinks enriched with calcium. - Plain fermented milk drinks.   E2: Combination drinks of no more than 330mls per portion:   - Combinations of fruit juice or vegetable juice with—   (a) plain water, in which case the fruit juice or vegetable juice must be at least 45% by volume, but no more than 150mls, and may contain vitamins and minerals;  (b) lower fat milk, lactose reduced milk or plain fermented milk drinks (in each case with or without plain water) in which case the milk or fermented milk drink must be at least 50% by volume and may contain vitamins, minerals and less than 5% added sugars or honey; lower fat milk” means milk the fat content of which has been reduced to not more than 1.8%;  (c) plain soya, rice or oat drink (in each case with or without plain water) in which case the soya, rice or oat drink must be at least 50% by volume and may contain vitamins, minerals and less than 5% added sugars or honey.   - Combinations of lower fat milk, lactose reduced milk, plain fermented milk drinks or plain soya, rice or oat drinks (in each case with or without plain water) with cocoa, in which case the lower fat milk, lactose reduced milk, fermented milk drink, soya, rice or oat drink must be at least 50% by volume and may contain vitamins, minerals and less than 5% added sugars or honey. - Flavoured lower fat milk, flavoured lactose reduced milk or flavoured soya, rice or oat drinks, in which case the lower fat milk, lactose reduced milk, soya, rice or oat drink must be at least 90% by volume and may contain vitamins, minerals and less than 5% added sugars or honey. - Tea and coffee. - Hot chocolate which may contain vitamins, minerals and less than 5% added sugars or honey.   Flavoured water cannot be provided in schools [3].  *The FUEL team includes the following as non-compliant: Flavoured water not made with fruit (must meet rule for combination drinks); Fizzy drinks not made with fruit (must meet rule for combination drinks); Diet drinks not made with fruit (must meet rule for combination drinks); Squashes; Self-serve drinks as volume is not controlled (except water or low fat milk)* |
| The supply of drinking water required by regulation 9(1) of the School Premises (England) Regulations 2012(1) must be provided free of charge at all times to registered pupils on the school premises [2] / Free, fresh drinking water at all times [1] | *The research team observe the presence of drinking water stations, which could include fountains, taps, jugs or cups (pre-filled or filled on request). It is noted that the research team may miss some drinking water facilities if not visible in areas observed during the visit.* |
| (1) Lower fat milk and lactose reduced milk (sub-group D1^g^) must be made available on every school day at a time during school hours [2] / Lower fat milk must be available for drinking at least once a day during school hours [1] | Lower fat milk means milk the fat content of which has been reduced to not more than 1.8% [2], which is the equivalent to semi-skimmed milk [3]. *The FUEL team includes flavoured milk in this standard.* |
| **SCHEDULE 4: REQUIREMENTS FOR FOOD PROVIDED OTHERWISE THAN AS PART OF A SCHOOL LUNCH [2]/ Standards for school food other than lunch [1]** |  |
| Either fruit or vegetables, or both fruit and vegetables must be available in any place on the school premises where food is provided [2]/ Fruit and/or vegetables available in all school food outlets [1] | *The FUEL team include all food outlets (including hatches / pods, outdoor kiosks, sixth form centres, cafes) and all occasions whether food is available, whether provided by the catering team or otherwise (includes breakfast, break time, lunch, after school before 6pm). The FUEL team includes fruits or vegetables provided as an accompaniment or within a composite dish.* |
| Savoury crackers and breadsticks must not be provided [2] / No savoury crackers or breadsticks [1] |  |
| Cakes and biscuits must not be provided. Desserts must not be provided, other than (a)yoghurt; or (b)fruit-based desserts (with a content of at least 50% fruit measured by volume of raw ingredients) [2]/ No cakes, biscuits, pastries or desserts (except yoghurt or fruit-based desserts containing at least 50% fruit) [1] | Cakes and biscuits include manufactured, bought-in products and prepared from scratch cakes and biscuits such as individual cakes, buns and pastries, scones, sweet and savoury biscuits [1]. The FUEL team include cakes, tray bakes, muffins, scones, doughnuts, biscuits and flapjack, as outlined in [1]. Croissants are included in this definition [3]. The following are permitted: malt loaf and other bread-type products such as bagels, currant and fruit bread, crumpets, tea cakes and English muffins [3]. *The FUEL team do not include teacakes or savoury pastries.*  Desserts include cereal and starch based desserts such as rice, semolina, tapioca pudding, fruit-based desserts such as jelly, fruit compote, crumbles, and sponge puddings, vegetable-based desserts such as soya-based mousse, egg-based desserts such as ready to eat products and products prepared from egg such as flans, egg custard, custard fillings in pies, meringues, dairy-based desserts such as puddings with dairy-based fillings, fruit or flavoured yoghurt or other fermented milk and/or milk products, ice cream, mousse and fat-based desserts, such as ice cream, made with vegetable fats [1]. *The FUEL team include jellies within this definition as outlined in [1].Do not include waffles and pancakes.* |
| **Other notes on application and interpretation** |  |
| Mealtimes / eating occasions out of scope of the SFS | 2) Nothing in these Regulations applies to food provided [2]—  (a)at parties or celebrations to mark religious or cultural occasions;  (b)at fund-raising events;  (c)as rewards for achievement, good behaviour or effort;  (d)for use in teaching food preparation and cookery skills, including where the food prepared is served to pupils as part of a school lunch; or  (e)on an occasional basis by parents or pupils. |

^a^A1: All types of bread, pasta, noodles, rice, potatoes, sweet potatoes, yams, millet and cornmeal

^b^A2: All types of bread with no added fat or oil

^c^B2: Vegetables of all types, whether fresh, frozen or dried. Vegetables canned in water or juice.

^d^B1: Fruit of all types, whether fresh, frozen or dried. Fruit canned in water or juice. Fruit-based desserts (with a content of at least 50% fruit measured by the weight of the raw ingredients).

^e^C: Meat and fish (in each case whether fresh, frozen, canned or dried), eggs, nuts, pulses and beans, other than green beans. Other non-dairy sources of protein. Any food containing meat together with food from groups A, B or D.

^f^D2: Cheese, yoghurt (including frozen), fromage frais and custard

^g^D1: Lower fat milk and lactose reduced milk.

.

Table B. How SFS were assessed, and judgements combined

| **School Food Standards** | **Observation** | **Menu** | **Summary judgement** |
| --- | --- | --- | --- |
| **Standards that apply across the whole school day** | **Assessed?** | **Assessed?** |  |
| Starchy foods cooked in fat and oil no more than two days a week (applies across the whole school day) | N | Y | Use menu only |
| A meat or poultry product (manufactured or homemade) no more than twice a week (applies across the whole school day) | Y | Y | If either the observation or menu features >2 items, rate as non-compliant. If the observation assessment features 1 or 2 items, refer to the menu to identify any additional items offered on a weekly basis that would result in non-compliance. |
| Lower fat milk and lactose reduced milk must be available for drinking at least once a day during school hours (applies across the whole school day) | Y | N | Use observation only^a^ |
| No more than two portions of food that have been deep-fried, batter-coated, or breadcrumb-coated, each week (applies across the whole school day) | Y | Y | If either the observation or menu features >2 items, rate as non-compliant. If the observation assessment features 1 or 2 items, refer to the menu to identify any additional items offered on a weekly basis that would result in non-compliance. |
| No more than two portions of food which include pastry each week (applies across the whole school day) | Y | Y | If either the observation or menu features >2 items, rate as non-compliant. If the observation assessment features 1 or 2 items, refer to the menu to identify any additional items offered on a weekly basis that would result in non-compliance. |
| No snacks, except nuts, seeds, vegetables and fruit with no added salt, sugar or fat (applies across the whole school day) | Y | N | Use observation only |
| No confectionery, chocolate or chocolate coated products (applies across the whole school day) | Y | If necessary | Use observation (if observation indicates compliance, check the menu for any non-compliant items) |
| Salt must not be available to add to food after it has been cooked (applies across the whole school day) | Y | N | Use observation only |
| Any condiments must be limited to sachets or portions of no more than 10 grams or one teaspoonful (applies across the whole school day) | Y | N | Use observation only |
| SFS-compliant drinks only (applies across the whole school day) | Y | N | Use observation only |
| Free fresh drinking water at all times (applies across the whole school day) | Y | N | Use observation only |
| Where dried fruit is provided it must have no more than 0.5% vegetable oil as a glazing agent. | Y | N | Use observation only |
| **Food provided outside of lunch** |  |  |  |
| Fruit and/or vegetables available at every outlet | Y | N | Use observation only^a^ |
| No savoury crackers or breadsticks | Y | N | Use observation only |
| No cakes or biscuits | Y | N | Use observation only |
| No desserts other than yoghurt or fruit-based desserts | Y | N | Use observation only |
| **Lunch time standards** |  |  |  |
| One or more portions of food from this group every day at lunch (Starchy food) | Y | Y | If menu features <1 portion per day, review observation assessment to identify any daily items not listed on the menu e.g. sandwiches^a^ |
| Bread with no added fat or oil available every day at lunch | Y | N | Use observation only^a^ |
| Three or more different starchy foods each week at lunch | If necessary | Y | Use menu assessment (if this indicates non-compliance, review observation assessment for ≥3 different starchy foods on day of observation) |
| One or more wholegrain varieties of starchy food each week at lunch | Y | Y | If either the observation or menu features a wholegrain item, judge as compliant^b^ |
| One or more portions of vegetables or salad as an accompaniment every day at lunch | Y | Y | If menu indicates non-compliance (<1 item per day), review observation assessment to identify any daily items not listed on the menu e.g. vegetables sticks, salad bar^a^ |
| One or more portions of fruit every day at lunch | Y | If necessary | If observation indicates non-compliance, review menu for any additional information e.g. fruit-based dessert |
| A dessert containing at least 50% fruit two or more times per week at lunch | N | Y | Use menu only |
| At least three different types of vegetables each week at lunch | Y | Y | If menu indicates non-compliance, review observation assessment for ≥3 different vegetables on day of observation |
| At least three different types of fruit each week at lunch | Y | Y | If menu indicates non-compliance, review observation assessment for ≥3 different fruits on day of observation |
| A portion of food from this group every day at lunch (Meat, fish, eggs, beans and other non-dairy sources of protein) | Y | Y | If menu features <1 portion per day, review observation assessment to identify any daily items not listed on the menu e.g. ham sandwich^a^ |
| A portion of meat or poultry on three or more days per week | N | Y | Use menu only |
| Oily fish once or more every three weeks | If necessary | Y | Use menu assessment (if menu indicates non-compliance, review the observation for any daily items not detailed in menu e.g. salmon sandwich, salmon salad)^c^ |
| For vegetarians, a portion of non-dairy protein on three or more days each week | N | Y | Use menu only |
| A portion of food from this group every day at lunch (Milk and dairy) | Y | Y | If menu features <1 portion per day, review observation assessment to identify any daily items not listed on the menu e.g. cheese sandwich^a^ |
| Desserts, cakes and biscuits are allowed at lunchtime. They must not contain any confectionery | Y | Y | If menu assessment indicates compliance, review observation for any daily items not detailed in menu e.g. chocolate chip cookie^a^ |
| Savoury crackers or breadsticks can be served at lunch with fruit or vegetables or dairy food | Y | N | Use observation only |

^a^Assumes a similar offering every day; ^b^Assumes a similar offering at least once every week; ^c^Assumes a similar offering at least once every three weeks

References

[1] School Food Plan (2014) School Food Standards. A practical guide for schools their cooks and caterers. Accessed 7/1/22. Available here: <https://www.schoolfoodplan.com/wp-content/uploads/2015/01/School-Food-Standards-Guidance-FINAL-V3.pdf>

[2] The Secretary of State for Education (2014) STATUTORY INSTRUMENTS 2014 No. 1603. The Requirements for School Food Regulations 2014. Accessed 7/1/22. Available here: <https://www.legislation.gov.uk/uksi/2014/1603/contents/made>

[3] School Food Plan (2014) School food standards regulations Q&A for consultation. Accessed 7/1/22. Available here: <http://www.schoolfoodplan.com/wp-content/uploads/2014/03/Standards-Consultation-QA-6-March-2014.pdf>
