# Supplementary material for: National school food standards in England: a cross-sectional study to explore compliance in secondary schools and impact on pupil nutritional intake
Source: Int J Behav Nutr Phys Act. 2024 Oct 24;21:123. doi: 10.1186/s12966-024-01672-w (PMC11515374; doi:10.1186/s12966-024-01672-w)
Supplement: Supplementary file 3 — Additional File 2: Process of assessment of school compliance with the school food standards (SFS) [file 12966_2024_1672_MOESM3_ESM.docx]

**Additional File 3: School Food Standards aiming to: A) increase dietary variety, and B) restrict high fat, sugar and energy-dense foods/drinks**

| **Standards grouping** | **Selected standards** |
| --- | --- |
| 1. Standards aiming to increase dietary variety | - Lower fat milk and lactose reduced milk must be available for drinking at least once a day during school hours (applies across the whole school day) - Fruit and/or vegetables available at every outlet outside of lunch - One or more portions of Starchy food every day at lunch - Three or more different starchy foods each week at lunch - One or more wholegrain varieties of starchy food each week at lunch - One or more portions of vegetables or salad as an accompaniment every day at lunch - One or more portions of fruit every day at lunch - A dessert containing at least 50% fruit two or more times per week at lunch - At least three different types of vegetables each week at lunch - At least three different types of fruit each week at lunch - A portion of meat, fish, eggs, beans and other non-dairy sources of protein every day at lunch - A portion of meat or poultry on three or more days per week at lunch - Oily fish once or more every three weeks at lunch - For vegetarians, a portion of non-dairy protein on three or more days each week at lunch - A portion of milk and dairy food every day at lunch |
| 1. Standards aiming to restrict high fat, sugar and energy-dense foods/drinks | - Starchy foods cooked in fat and oil no more than two days a week (applies across the whole school day) - No more than two portions of food that have been deep-fried, batter-coated, or breadcrumb-coated, each week (applies across the whole school day) - No more than two portions of food which include pastry each week (applies across the whole school day) - No snacks, except nuts, seeds, vegetables and fruit with no added salt, sugar or fat (applies across the whole school day) - No confectionery, chocolate or chocolate coated products (applies across the whole school day) - Any condiments must be limited to sachets or portions of no more than 10 grams or one teaspoonful (applies across the whole school day) - SFS-compliant drinks only (applies across the whole school day) - Free fresh drinking water at all times (applies across the whole school day) - No cakes or biscuits outside of lunch - No desserts other than yoghurt or fruit-based desserts outside of lunch - Bread with no added fat or oil available every day at lunch - Desserts, cakes and biscuits are allowed at lunchtime. They must not contain any confectionery |
| 1. Standards that do not contribute to types A and B above | - A meat or poultry product (manufactured or homemade) no more than twice a week (applies across the whole school day) - Salt must not be available to add to food after it has been cooked (applies across the whole school day) - Where dried fruit is provided it must have no more than 0.5% vegetable oil as a glazing agent - No savoury crackers or breadsticks outside of lunch - Savoury crackers or breadsticks can be served at lunch with fruit or vegetables or dairy food |
