# Supplementary material for: National school food standards in England: a cross-sectional study to explore compliance in secondary schools and impact on pupil nutritional intake
Source: Int J Behav Nutr Phys Act. 2024 Oct 24;21:123. doi: 10.1186/s12966-024-01672-w (PMC11515374; doi:10.1186/s12966-024-01672-w)
Supplement: Supplementary file 4 — Additional File 3: School Food Standards aiming to: (A) increase dietary variety, and (B) restrict high fat, sugar and energy-dense foods/drinks [file 12966_2024_1672_MOESM4_ESM.docx]

**Additional File 4: List of foods added to Intake24 to increase applicability to a culturally diverse population**

| **Cultural community** | **Food item** |
| --- | --- |
| South Asian | Bhatura |
|  | Channa |
|  | Dahi vada / Dahiwada |
|  | Dhokla |
|  | Fafda |
|  | Farsi poori |
|  | Gulab jamun |
|  | Idly |
|  | Jalebi |
|  | Kachori |
|  | Kadhi with pakories |
|  | Katlama, white |
|  | Katlama, wholemeal (brown) |
|  | Kheer |
|  | Khichdi |
|  | Khudi |
|  | Ladoo / Laddu |
|  | Mithai / Burfi |
|  | Mutton |
|  | Pawpaw |
|  | Payasam |
|  | Poori |
|  | Sev / Ganthia |
|  | Shami kebab |
|  | Shrikhand |
| West African | Agege bread |
|  | Amala |
|  | Ayamashe |
|  | Banga / Ofe Akwu / Palm fruit soup |
|  | Bream / Brim, fried |
|  | Callaloo and chocho |
|  | Chocho / Chayote |
|  | Cornmeal porridge |
|  | Eddoe / Dasheen / Tannia |
|  | Edikang ikong soup |
|  | Efo riro soup |
|  | Egusi / Melon seed soup |
|  | Ewa agoyin / Ewa G / beans and pepper stew |
|  | Ewedu soup |
|  | Fufu |
|  | Gbegiri soup |
|  | Goat meat |
|  | Green banana porridge |
|  | Iwisa |
|  | Jollof rice |
|  | Kenkey |
|  | Njeera |
|  | Ofe onugbu / Bitter leaf soup |
|  | Patties |
|  | Rice and pigeon peas |
|  | Salt fish, boiled |
|  | Tilapia, fried |
|  | West Indian soup |
| East Asian | Bell fruit |
|  | Kai lan |
|  | Longan |
| Middle Eastern | Fattoush |
|  | Hummus fatteh |
|  | Lamb tagine |
|  | Muttabal |
|  | Tarator |
| Other | Lentil bolognaise |
|  | Pierogi |
