# Supplementary material for: National school food standards in England: a cross-sectional study to explore compliance in secondary schools and impact on pupil nutritional intake
Source: Int J Behav Nutr Phys Act. 2024 Oct 24;21:123. doi: 10.1186/s12966-024-01672-w (PMC11515374; doi:10.1186/s12966-024-01672-w)
Supplement: Supplementary file 5 — Additional File 4: Foods commonly consumed by minority ethnic communities that were identified and included in the Intake24 dietary recall tool [file 12966_2024_1672_MOESM5_ESM.docx]

**Additional File 5: Relationship between the percentage of school food standards (SFS) complied with and school Income Deprivation Affecting Children Index (IDACI)**


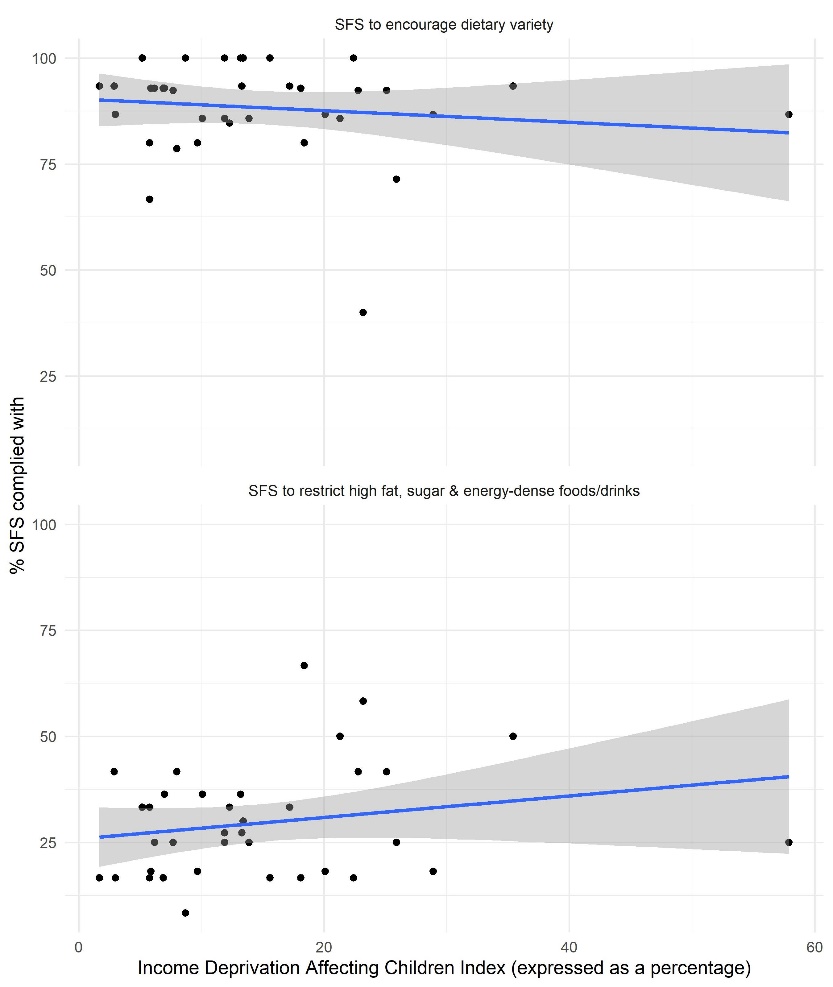

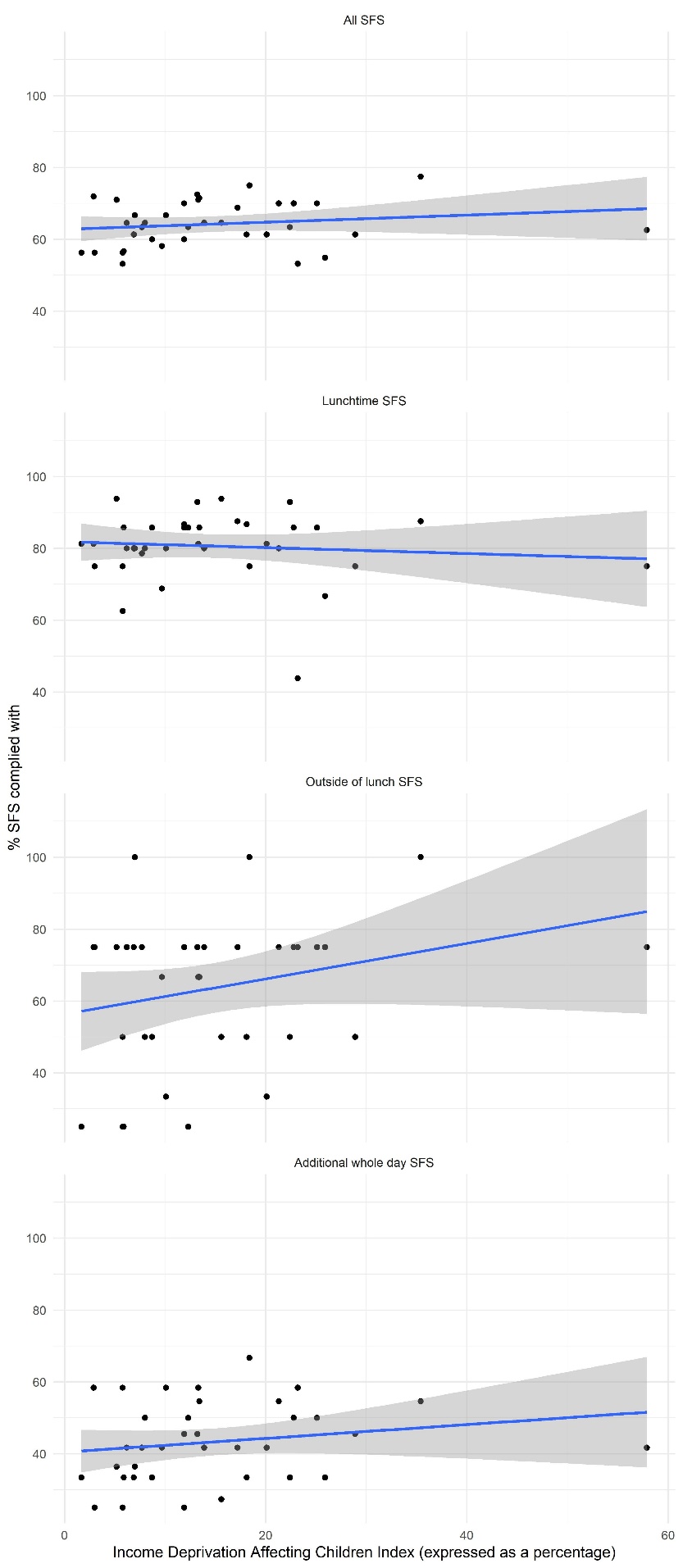
**A:** All SFS and SFS categories **B:** SFS types
