# Supplementary material for: National school food standards in England: a cross-sectional study to explore compliance in secondary schools and impact on pupil nutritional intake
Source: Int J Behav Nutr Phys Act. 2024 Oct 24;21:123. doi: 10.1186/s12966-024-01672-w (PMC11515374; doi:10.1186/s12966-024-01672-w)
Supplement: Supplementary file 6 — Additional File 5: Relationship between the percentage of school food standards (SFS) complied with and school Income Deprivation Affecting Children Index (IDACI) [file 12966_2024_1672_MOESM6_ESM.docx]

**Additional File 6: Numbers and % of schools meeting each school food standard (SFS)**

| **Standards** |  | **Schools meeting the standard N (%)** | | |
| --- | --- | --- | --- | --- |
| **Lunch time standards** | **Schools providing data** | **All schools** | **SFS-mandated** | **SFS-non-mandated** |
| One or more portions of starchy foods every day | 36 | 36 (100.0) | 13 (100.0) | 23 (100.0) |
| Bread with no added fat or oil available every day | 35 | 10 (28.6) | 4 (33.3) | 6 (26.1) |
| Three or more different starchy foods each week | 36 | 35 (97.2) | 12 (92.3) | 23 (100.0) |
| One or more wholegrain varieties of starchy food each week | 36 | 29 (80.5) | 10 (76.9) | 19 (82.6) |
| One or more portions of vegetables/salad as an accompaniment every day | 36 | 36 (100.0) | 13 (100.0) | 23 (100.0) |
| One or more portions of fruit every day | 36 | 36 (100.0) | 13 (100.0) | 23 (100.0) |
| A dessert containing at least 50% fruit two or more times per week | 18 | 5 (27.8) | 1 (25.0) | 4 (28.6) |
| At least three different types of vegetables each week | 36 | 36 (100.0) | 13 (100.0) | 23 (100.0) |
| At least three different types of fruit each week | 36 | 32 (88.9) | 11 (84.6) | 21 (91.3) |
| A portion of meat, fish, eggs, beans and other non-dairy sources of protein food every day | 36 | 35 (97.2) | 12 (92.3) | 23 (100.0) |
| A portion of meat or poultry on three or more days per week | 36 | 36 (100.0) | 13 (100.0) | 23 (100.0) |
| Oily fish once or more every three weeks | 27 | 10 (37.0) | 4 (50.0) | 6 (31.6) |
| For vegetarians, a portion of non-dairy protein on three or more days each week | 35 | 33 (94.3) | 12 (92.3) | 21 (95.4) |
| A portion of milk or dairy foods every day | 36 | 35 (97.2) | 12 (92.3) | 23 (100.0) |
| Desserts, cakes and biscuits are allowed at lunchtime. They must not contain any confectionery | 35 | 3 (8.6) | 0 (0.0) | 3 (13.6) |
| Savoury crackers or breadsticks can be served at lunch with fruit or vegetables or dairy food^a^ | 36 | 32 (88.9) | 12 (92.3) | 20 (86.9) |
| **Standards that apply to food provided outside of lunch** |  |  |  |  |
| Fruit and/or vegetables available at every outlet | 36 | 29 (80.5) | 9 (69.2) | 20 (86.9) |
| No savoury crackers or breadsticks^a^ | 36 | 33 (91.6) | 13 (100.0) | 20 (86.9) |
| No cakes or biscuits | 36 | 6 (16.6) | 4 (30.7) | 2 (8.7) |
| No desserts other than yoghurt or fruit-based desserts | 31 | 21 (67.7) | 8 (66.6) | 13 (68.4) |
| No more than two portions of food which include pastry each week | 35 | 5 (14.3) | 3 (23.1) | 2 (9.1) |
| No snacks, except nuts, seeds, vegetables and fruit with no added salt, sugar or fat | 36 | 18 (50.0) | 7 (53.8) | 11 (47.8) |
| No confectionery, chocolate or chocolate coated products | 36 | 2 (5.5) | 0 (0.0) | 2 (8.7) |
| Where dried fruit is provided it must have no more than 0.5% vegetable oil as a glazing agent^a^ | 32 | 31 (96.9) | 11 (100.00) | 20 (95.2) |
| Salt must not be available to add to food after it has been cooked | 35 | 32 (91.4) | 12 (92.3) | 20 (90.9) |
| Any condiments must be limited to sachets or portions of no more than 10 grams or one teaspoonful | 34 | 17 (50.0) | 5 (41.6) | 12 (54.5) |
| Compliant drinks only | 36 | 5 (13.9) | 2 (15.4) | 3 (13.0) |
| Free fresh drinking water at all times | 35 | 34 (97.1) | 12 (92.3) | 22 (100.0) |
