# Supplementary material for: National school food standards in England: a cross-sectional study to explore compliance in secondary schools and impact on pupil nutritional intake
Source: Int J Behav Nutr Phys Act. 2024 Oct 24;21:123. doi: 10.1186/s12966-024-01672-w (PMC11515374; doi:10.1186/s12966-024-01672-w)
Supplement: Supplementary file 7 — Additional File 6: Numbers and % of schools meeting each school food standard (SFS) [file 12966_2024_1672_MOESM7_ESM.docx]

**Additional File 7: Pupils’ nutrient and food intakes on a school day: at lunch, across the whole school day, and during 24-hours - associations with SFS-mandated/non-mandated school status, additionally adjusted for energy intake**

| **Nutritional outcome** | **Mean difference (MD) or incidence rate ratio (IRR) for SFS-mandated schools, compared with SFS-non-mandated schools** | | | | | |
| --- | --- | --- | --- | --- | --- | --- |
|  | **Lunch** (n=1,878) | | **School day** (n=1,934) | | **24 hours** (n=2,045) | |
|  | MD/IRR (95% CI) | p value | MD/IRR (95% CI) | p value | MD/IRR (95% CI) | p value |
| **Free sugar (g)**; MD | -1.13 (-2.89, 0.64) | 0.21 | 1.55 (-1.4, 4.51) | 0.31 | 4.78 (-1.31, 10.87) | 0.12 |
| **Fat (g)**; MD | 0.31 (-0.30, 0.92) | 0.32 | -0.01 (-0.80, 0.77) | 0.98 | 0.25 (-1.13, 1.63) | 0.72 |
| **Fibre (g)**; MD | 0.15 (-0.10, 0.41) | 0.24 | 0.04 (-0.29, 0.38) | 0.79 | -0.38 (-1.12, 0.36) | 0.31 |
| **F&V portions**; MD | **-0.20 (-0.32, -0.08)** | **0.001** | **-0.19 (-0.31, -0.06)** | **0.005** | **-0.29 (-0.56, -0.02)** | **0.03** |
| **SSB items**; IRR | 1.15 (0.87, 1.52) | 0.32 | 1.16 (0.85, 1.59) | 0.35 | **1.32 (1.03, 1.69)** | **0.03** |
| **HFSS items**; IRR | 0.99 (0.89, 1.10) | 0.82 | 0.99 (0.91, 1.07) | 0.73 | 1.02 (0.94, 1.10) | 0.69 |
| **Sugar/chocolate confectionery items**; IRR | 1.08 (0.84, 1.38) | 0.56 | **1.25 (1.04, 1.50)** | **0.02** | 1.08 (0.95, 1.23) | 0.24 |

SFS = School Food Standards; CI = Confidence Interval; F&V = Fruit and Vegetable; SSB = Sugar Sweetened Beverage; HFSS = High Fat Sugar Salt.

Model covariates: Energy intake, sex, age, ethnicity, Index of Multiple Deprivation (IMD) quintile group, lunch source, school % Free School Meals (%FSM), school Income Deprivation Affecting Children Index (IDACI), school six form, school catering provision, school religious status, data collection year, year group.
