# Supplementary material for: National school food standards in England: a cross-sectional study to explore compliance in secondary schools and impact on pupil nutritional intake
Source: Int J Behav Nutr Phys Act. 2024 Oct 24;21:123. doi: 10.1186/s12966-024-01672-w (PMC11515374; doi:10.1186/s12966-024-01672-w)
Supplement: Supplementary file 8 — Additional File 7: Pupils’ nutrient and food intakes on a school day: at lunch, across the whole school day, and during 24-hours - associations with SFS-mandated/non-mandated school status, additionally adjusted for energy intake [file 12966_2024_1672_MOESM8_ESM.docx]

**Additional File 8: Interaction effects in the models to explore differences in pupil nutritional intakes between schools mandated or not mandated to comply with the school food standards (SFS)**

Table A. Pupil nutritional outcome models: adjusted coefficients for SFS-mandated status-lunch source (100% school-provided) interaction terms

| **Outcome** | **Lunch**  **Coefficient (95% CI), p value** | **School day**  **Coefficient (95% CI), p value** | **24-hour day**  **Coefficient (95% CI), p value** |
| --- | --- | --- | --- |
| Free sugar | 0.007 (-3.52, 3.53) p = 0.997 | -1.35 (-6.75, 4.06) p = 0.63 | -4.06 (-14.23, 6.11) p = 0.43 |
| % energy intake from free sugar | -1.86 (-4.44, 0.72) p = 0.16 | -2.16 (-4.55, 0.22) p = 0.08 | -1.34 (-2.96, 0.27) p = 0.10 |
| Energy intake | 52.13 (-7.00, 111.25) p = 0.08 | 38.33 (-42.05, 118.71) p = 0.35 | 3.25 (-146.06, 152.56) p = 0.97 |
| Fat intake | 2.62 (-0.32, 5.56) p = 0.08 | 1.67 (-2.17, 5.50) p = 0.39 | -0.62 (-7.46, 6.22) p = 0.86 |
| Fibre intake | 0.46 (-0.14, 1.05) p = 0.13 | 0.56 (-0.17, 1.30) p = 0.13 | 0.57 (-0.86, 2.00) p = 0.43 |
| F&V portions | 0.15 (-0.04, 0.34) p = 0.124 | 0.15 (-0.08, 0.37) p = 0.20 | 0.28 (-0.11, 0.66) p = 0.16 |
| Number of SSB | 0.72 (0.43, 1.22) p = 0.22 | 0.92 (0.57, 1.48) p = 0.74 | 0.81 (0.59, 1.10) p = 0.18 |
| Number of HFSS items | 1.05 (0.87, 1.28) p = 0.59 | 0.98 (0.84, 1.15) p = 0.83 | 0.97 (0.86, 1.09) p = 0.58 |
| Number of confectionery items | 1.25 (0.60, 2.59) p = 0.55 | 1.03 (0.68, 1.57) p = 0.88 | 0.88 (0.66, 1.17) p = 0.37 |

**Note:** The coefficients shown for Sugar-Sweetened Beverages (SSB), High fat, sugar, salt (HFSS) items and confectionery items are Incidence Rate Ratios (IRR) as Poisson models were used

Table B. Pupil nutritional outcome models: adjusted coefficients for SFS-mandated status-IMD quintile group interaction terms

| **Outcome** | **Interaction term** | **Lunch**  **Coefficient (95% CI), p value** | **School day**  **Coefficient (95% CI), p value** | **24-hour day**  **Coefficient (95% CI), p value** |
| --- | --- | --- | --- | --- |
| Free sugar | SFS-IMD Quintile 2 | -2.70 (-8.28, 2.88) p = 0.34 | -2.32 (-10.92, 6.28) p = 0.60 | -5.56 (-22.18, 11.06) p = 0.51 |
|  | SFS-IMD Quintile 3 | -2.46 (-8.03, 3.12) p = 0.39 | -3.38 (-12.11, 5.34) p = 0.45 | -11.96 (-28.70, 4.77) p = 0.16 |
|  | SFS-IMD Quintile 4 | -1.80 (-8.04, 4.44) p = 0.57 | -1.55 (-11.27, 8.16) p = 0.75 | -1.64 (-20.50, 17.23) p = 0.87 |
|  | SFS-IMD Quintile 5 | -0.98 (-7.25, 5.29) p = 0.76 | 0.53 (-9.29, 10.34) p = 0.92 | 17.14 (-1.88, 36.16) p = 0.08 |
| % energy intake from free sugar | SFS-IMD Quintile 2 | -1.89 (-5.92, 2.14) p = 0.36 | -1.63 (-5.36, 2.11) p = 0.39 | 0.89 (-1.71, 3.50) p = 0.50 |
|  | SFS-IMD Quintile 3 | -3.21 (-7.34, 0.93) p = 0.13 | -2.76 (-6.61, 1.10) p = 0.16 | -0.74 (-3.43, 1.95) p = 0.59 |
|  | SFS-IMD Quintile 4 | -1.35 (-5.94, 3.24) p = 0.56 | -1.33 (-5.59, 2.94) p = 0.54 | 0.31 (-2.70, 3.32) p = 0.84 |
|  | SFS-IMD Quintile 5 | 1.67 (-2.98, 6.32) p = 0.48 | 1.80 (-2.54, 6.13) p = 0.42 | **3.57 (0.51, 6.63) p = 0.02** |
| Energy intake | SFS-IMD Quintile 2 | -5.80 (-98.98, 87.38) p = 0.90 | -20.98 (-149.23, 107.27) p = 0.75 | -92.40 (-334.90, 150.10) p = 0.46 |
|  | SFS-IMD Quintile 3 | 42.30 (-51.57, 136.17) p = 0.38 | -0.24 (-129.37, 128.89) p = 0.997 | -93.83 (-340.17, 152.51) p = 0.46 |
|  | SFS-IMD Quintile 4 | 32.72 (-72.11, 137.55) p = 0.54 | 11.39 (-132.70, 155.49) p = 0.88 | -90.92 (-367.84, 185.99) p = 0.52 |
|  | SFS-IMD Quintile 5 | -66.67 (-172.25, 38.91) p = 0.22 | -67.97 (-213.02, 77.09) p = 0.36 | -28.12 (-308.31, 252.06) p = 0.84 |
| Fat intake | SFS-IMD Quintile 2 | -0.78 (-5.37, 3.81) p = 0.74 | -0.005 (-6.06, 6.05) p = 0.999 | -6.10 (-16.92, 4.71) p = 0.27 |
|  | SFS-IMD Quintile 3 | 1.80 (-2.82, 6.41) p = 0.45 | 0.47 (-5.64, 6.57) p = 0.88 | -2.47 (-13.42, 8.49) p = 0.66 |
|  | SFS-IMD Quintile 4 | 2.96 (-2.20, 8.11) p = 0.26 | 2.36 (-4.44, 9.17) p = 0.50 | -5.25 (-17.55, 7.05) p = 0.40 |
|  | SFS-IMD Quintile 5 | -1.57 (-6.75, 3.61) p = 0.55 | -1.38 (-8.23, 5.48) p = 0.69 | -4.55 (-16.97, 7.88) p = 0.47 |
| Fibre intake | SFS-IMD Quintile 2 | 0.07 (-0.86, 1.00) p = 0.88 | -0.03 (-1.19, 1.13) p = 0.96 | 0.30 (-2.01, 2.61) p = 0.80 |
|  | SFS-IMD Quintile 3 | 0.29 (-0.67, 1.24) p = 0.56 | -0.24 (-1.43, 0.95) p = 0.69 | -0.93 (-3.32, 1.46) p = 0.45 |
|  | SFS-IMD Quintile 4 | -0.12 (-1.18, 0.95) p = 0.83 | -0.44 (-1.76, 0.88) p = 0.51 | -1.13 (-3.81, 1.54) p = 0.42 |
|  | SFS-IMD Quintile 5 | -0.61 (-1.68, 0.47) p = 0.27 | -0.80 (-2.14, 0.53) p = 0.24 | -0.19 (-2.91, 2.54) p = 0.89 |
| F&V portions | SFS-IMD Quintile 2 | -0.03 (-0.33, 0.27) p = 0.85 | -0.01 (-0.37, 0.34) p = 0.93 | 0.12 (-0.50, 0.73) p = 0.71 |
|  | SFS-IMD Quintile 3 | -0.06 (-0.37, 0.24) p = 0.68 | -0.20 (-0.55, 0.16) p = 0.28 | -0.10 (-0.73, 0.54) p = 0.76 |
|  | SFS-IMD Quintile 4 | -0.10 (-0.44, 0.24) p = 0.56 | -0.11 (-0.51, 0.28) p = 0.57 | 0.17 (-0.54, 0.88) p = 0.64 |
|  | SFS-IMD Quintile 5 | -0.09 (-0.43, 0.25) p = 0.61 | -0.06 (-0.46, 0.35) p = 0.79 | 0.17 (-0.56, 0.89) p = 0.65 |
| Number of SSB | SFS-IMD Quintile 2 | 1.04 (0.50, 2.17) p = 0.92 | 1.39 (0.68, 2.86) p = 0.37 | 1.15 (0.70, 1.90) p = 0.57 |
|  | SFS-IMD Quintile 3 | 0.86 (0.40, 1.88) p = 0.71 | 1.30 (0.61, 2.75) p = 0.50 | 0.83 (0.49, 1.39) p = 0.48 |
|  | SFS-IMD Quintile 4 | 0.87 (0.36, 2.11) p = 0.76 | 1.05 (0.44, 2.50) p = 0.92 | 0.94 (0.52, 1.68) p = 0.83 |
|  | SFS-IMD Quintile 5 | 1.62 (0.67, 3.95) p = 0.29 | 2.01 (0.86, 4.68) p = 0.11 | 1.82 (1.00, 3.29) p = 0.05 |
| Number of HFSS items | SFS-IMD Quintile 2 | 1.12 (0.84, 1.48) p = 0.44 | 1.12 (0.89, 1.41) p = 0.35 | 1.12 (0.93, 1.33) p = 0.23 |
|  | SFS-IMD Quintile 3 | 1.08 (0.82-, .43) p = 0.58 | 1.05 (0.83, 1.33) p = 0.67 | 0.98 (0.82, 1.18) p = 0.83 |
|  | SFS-IMD Quintile 4 | 1.10 (0.82, 1.49) p = 0.52 | 1.06 (0.82, 1.36) p = 0.66 | 1.04 (0.85, 1.28) p = 0.67 |
|  | SFS-IMD Quintile 5 | 1.02 (0.75, 1.40) p = 0.90 | 1.11 (0.86, 1.44) p = 0.43 | 1.09 (0.89, 1.34) p = 0.42 |
| Number of confectionery items | SFS-IMD Quintile 2 | 0.96 (0.44, 2.09) p = 0.91 | 1.15 (0.66, 2.01) p = 0.63 | 1.35 (0.88, 2.06) p = 0.17 |
|  | SFS-IMD Quintile 3 | 0.70 (0.32, 1.50) p = 0.36 | 0.81 (0.47, 1.42) p = 0.47 | 1.01 (0.66, 1.54) p = 0.97 |
|  | SFS-IMD Quintile 4 | 0.78 (0.35, 1.75) p = 0.55 | 0.95 (0.52, 1.73) p = 0.86 | 1.32 (0.82, 2.10) p = 0.25 |
|  | SFS-IMD Quintile 5 | 0.87 (0.38, 1.99) p = 0.75 | 0.90 (0.49, 1.65) p = 0.73 | 1.25 (0.79, 1.97) p = 0.33 |

**Note:** The coefficients shown for Sugar-Sweetened Beverages (SSB), High fat, sugar, salt (HFSS) items and confectionery items as Poisson models were used

Table C. Pupil nutritional outcome models: adjusted coefficients for SFS-mandated status-Year group interaction terms

| **Outcome** | **Interaction term** | **Lunch**  **Coefficient (95% CI), p value** | **School day**  **Coefficient (95% CI), p value** | **24-hour day**  **Coefficient (95% CI), p value** |
| --- | --- | --- | --- | --- |
| Free sugar | SFS-Year 9 | 0.56 (-3.87, 4.99) p = 0.81 | -1.25 (-8.07, 5.57) p = 0.72 | 3.66 (-9.71, 17.03) p = 0.59 |
|  | SFS-Year 10 | 1.02 (-3.42, 5.45) p = 0.65 | -2.85 (-9.70, 3.99) p = 0.41 | -4.13 (-17.50, 9.24) p = 0.55 |
| % energy intake from free sugar | SFS-Year 9 | 1.95 (-1.20, 5.09) p = 0.23 | 0.08 (-2.84, 3.01) p = 0.96 | -0.54 (-2.60, 1.52) p = 0.61 |
|  | SFS-Year 10 | 1.92 (-1.23, 5.07) p = 0.23 | 0.23 (-2.71, 3.17) p = 0.88 | -0.75 (-2.81, 1.32) p = 0.48 |
| Energy intake | SFS-Year 9 | -25.12 (-98.78, 48.54) p = 0.50 | -20.14 (-122.27, 81.99) p = 0.70 | 108.36 (-85.13, 301.86) p = 0.27 |
|  | SFS-Year 10 | -12.49 (-86.22, 61.23) p = 0.74 | -78.92 (-181.41, 23.58) p = 0.13 | -54.26 (-247.77, 139.24) p = 0.58 |
| Fat intake | SFS-Year 9 | -0.97 (-4.60, 2.66) p = 0.60 | -0.34 (-5.16, 4.48) p = 0.89 | 7.27 (-1.37, 15.92) p = 0.10 |
|  | SFS-Year 10 | -0.56 (-4.20, 3.07) p = 0.76 | -3.31 (-8.14, 1.53) p = 0.18 | -2.34 (-10.98, 6.31) p = 0.60 |
| Fibre intake | SFS-Year 9 | -0.71 (-1.43, 0.02) p = 0.06 | -0.42 (-1.33, 0.50) p = 0.37 | 0.44 (-1.38, 2.27) p = 0.63 |
|  | SFS-Year 10 | -0.29 (-1.01, 0.44) p = 0.44 | -0.66 (-1.58, 0.26) p = 0.16 | -0.88 (-2.70, 0.95) p = 0.35 |
| F&V portions | SFS-Year 9 | -0.17 (-0.40, 0.07) p = 0.16 | -0.11 (-0.39, 0.17) p = 0.45 | 0.03 (-0.45, 0.52) p = 0.90 |
|  | SFS-Year 10 | 0.09 (-0.14, 0.33) p = 0.43 | 0.07 (-0.21, 0.35) p = 0.63 | -0.13 (-0.61, 0.36) p = 0.61 |
| Number of SSB | SFS-Year 9 | 1.09 (0.60, 2.00) p = 0.78 | 1.32 (0.75, 2.33) p = 0.34 | 1.27 (0.86, 1.87) p = 0.23 |
|  | SFS-Year 10 | 1.34 (0.72, 2.50) p = 0.35 | 1.22 (0.67, 2.22) p = 0.51 | 1.30 (0.87, 1.95) p = 0.21 |
| Number of HFSS items | SFS-Year 9 | 0.92 (0.74, 1.14) p = 0.46 | 0.90 (0.75, 1.08) p = 0.25 | 0.96 (0.84, 1.11) p = 0.59 |
|  | SFS-Year 10 | 1.01 (0.82, 1.25) p = 0.92 | 0.96 (0.80, 1.15) p = 0.67 | 0.99 (0.86, 1.14) p = 0.92 |
| Number of confectionery items | SFS-Year 9 | 0.79 (0.44, 1.43) p = 0.43 | 0.75 (0.49, 1.16) p = 0.20 | 0.89 (0.64, 1.24) p = 0.48 |
|  | SFS-Year 10 | 1.03 (0.57, 1.84) p = 0.92 | 0.96 (0.63, 1.47) p = 0.84 | 0.80 (0.57, 1.12) p = 0.19 |

**Note:** The coefficients shown for Sugar-Sweetened Beverages (SSB), High fat, sugar, salt (HFSS) items and confectionery items are IRRs as Poisson models were used
