# Supplementary material for: National school food standards in England: a cross-sectional study to explore compliance in secondary schools and impact on pupil nutritional intake
Source: Int J Behav Nutr Phys Act. 2024 Oct 24;21:123. doi: 10.1186/s12966-024-01672-w (PMC11515374; doi:10.1186/s12966-024-01672-w)
Supplement: Supplementary file 9 — Additional File 8: Interaction effects in the models to explore differences in pupil nutritional intakes between schools mandated or not mandated to comply with the school food standards (SFS) [file 12966_2024_1672_MOESM9_ESM.docx]

Additional File 9: Models to explore differences in pupil nutritional intakes between schools mandated or not mandated to comply with the school food standards (SFS) – sensitivity analyses

Table A. Use of imputed vs. original Index of Multiple Deprivation (IMD) variable as a coefficient in the model with the nutritional outcome of free sugar intake (g) at lunch

|  | **Model coefficient with original IMD variable** | **Model coefficient with imputed IMD variable** |
| --- | --- | --- |
| Pupils | 1,878 | 2,100 |
| Schools | 36 | 36 |
| SFS Status: Mandated | **-2.78 (-4.66, -0.90) p = 0.004** | **-2.03 (-3.76, -0.30) p = 0.02** |
| Sex: Male | -0.21 (-2.00, 1.59) p = 0.82 | -0.03 (-1.67, 1.62) p = 0.97 |
| Sex: Other | 0.36 (-4.63, 5.36) p = 0.89 | 0.63 (-3.93, 5.20) p = 0.79 |
| Age (years) | -1.13 (-3.67, 1.42) p = 0.39 | -0.16 (-2.49, 2.17) p = 0.89 |
| Ethnicity: Asian/Asian British | -1.69 (-4.12, 0.74) p = 0.17 | -1.16 (-3.41, 1.08) p = 0.31 |
| Ethnicity: Black/African/Caribbean/Black British | 1.71 (-2.31, 5.73) p = 0.40 | 2.10 (-1.54, 5.73) p = 0.26 |
| Ethnicity: Mixed/Multiple | -3.04 (-6.80, 0.73) p = 0.11 | -2.78 (-6.20, 0.65) p = 0.11 |
| Ethnicity: Other | 2.57 (-2.35, 7.49) p = 0.31 | 1.47 (-2.91, 5.85) p = 0.51 |
| IMD Quintile: 2 | 2.29 (-0.53, 5.11) p = 0.11 | 1.32 (-1.28, 3.92) p = 0.32 |
| IMD Quintile: 3 | 1.41 (-1.47, 4.30) p = 0.34 | 1.21 (-1.49, 3.90) p = 0.38 |
| IMD Quintile: 4 | 0.87 (-2.19, 3.92) p = 0.58 | 0.61 (-2.22, 3.43) p = 0.68 |
| IMD Quintile: 5 | 0.32 (-2.73, 3.37) p = 0.84 | 0.79 (-2.09, 3.68) p = 0.59 |
| Source of Lunch: 100% School Provided | **-3.55 (-5.18, -1.91) p < 0.001** | **-2.04 (-3.54, -0.54) p = 0.008** |
| Percent FSM | 0.07 (-0.04, 0.18) p = 0.21 | 0.03 (-0.07, 0.13) p = 0.57 |
| School Level IDACI | **0.12 (0.02, 0.23) p = 0.03** | **0.15 (0.05, 0.25) p = 0.003** |
| Sixth Form | **-3.18 (-5.14, -1.21) p = 0.002** | **-3.11 (-4.92, -1.29) p = 0.001** |
| Provision: External | 1.12 (-0.67, 2.91) p = 0.22 | 0.92 (-0.73, 2.56) p = 0.27 |
| Religious: Secular | **5.01 (1.88, 8.14) p = 0.002** | **5.04 (2.13, 7.96) p = 0.001** |
| Year collected: 2020/2021 | **-3.51 (-6.77, -0.25) p = 0.04** | **-4.08 (-7.09, -1.07) p = 0.008** |
| Year collected: 2021/2022 | 0.49 (-1.52, 2.51) p = 0.63 | 0.18 (-1.67, 2.04) p = 0.85 |
| Year: 9 | 0.88 (-4.61, 6.37) p = 0.75 | -2.16 (-7.17, 2.85) p = 0.40 |
| Year: 10 | 0.94 (-6.97, 8.86) p = 0.82 | -3.27 (-10.52, 3.98) p = 0.38 |

Table B. Use of imputed vs. original Age variable as a coefficient in the model with the nutritional outcome of free sugar intake (g) at lunch

|  | **Model coefficient with original Age variable** | **Model coefficient with imputed Age variable** |
| --- | --- | --- |
| Pupils | 1,878 | 1,913 |
| Schools | 36 | 36 |
| SFS Status: Mandated | **-2.78 (-4.66, -0.90) p =0.004** | **-2.88 (-4.75, -1.02) p =0.003** |
| Sex: Male | -0.21 (-2.00, 1.59) p =0.82 | 0.08 (-1.70, 1.86) p =0.93 |
| Sex: Other | 0.36 (-4.63, 5.36) p =0.88 | 0.34 (-4.60, 5.28) p =0.89 |
| Age (years) | -1.13 (-3.67, 1.42) p =0.39 | -1.09 (-3.63, 1.45) p =0.40 |
| Ethnicity: Asian/Asian British | -1.69 (-4.12, 0.74) p =0.17 | -1.87 (-4.27, 0.53) p =0.13 |
| Ethnicity: Black/African/Caribbean/Black British | 1.71 (-2.31, 5.73) p =0.40 | 1.58 (-2.44, 5.59) p =0.44 |
| Ethnicity: Mixed/Multiple | -3.04 (-6.80, 0.73) p =0.11 | -3.17 (-6.91, 0.57) p =0.10 |
| Ethnicity: Other | 2.57 (-2.35, 7.49) p =0.31 | 2.35 (-2.51, 7.20) p =0.34 |
| IMD Quintile: 2 | 2.29 (-0.53, 5.11) p =0.11 | 2.07 (-0.73, 4.87) p =0.15 |
| IMD Quintile: 3 | 1.41 (-1.47, 4.30) p =0.34 | 1.15 (-1.71, 4.01) p =0.43 |
| IMD Quintile: 4 | 0.87 (-2.19, 3.92) p =0.58 | 0.75 (-2.28, 3.79) p =0.63 |
| IMD Quintile: 5 | 0.32 (-2.73, 3.37) p =0.84 | 0.33 (-2.70, 3.35) p =0.83 |
| Source of Lunch: 100% School Provided | **-3.55 (-5.18, -1.91) p < 0.001** | **-3.51 (-5.13, -1.89) p < 0.001** |
| Percent FSM | 0.07 (-0.04, 0.18) p =0.20 | 0.07 (-0.04, 0.18) p =0.19 |
| School Level IDACI | **0.12 (0.02, 0.23) p =0.03** | **0.12 (0.01, 0.22) p =0.03** |
| Sixth Form | **-3.18 (-5.14, -1.21) p =0.002** | **-3.17 (-5.11, -1.23) p =0.001** |
| Provision: External | 1.12 (-0.67, 2.91) p =0.22 | 1.09 (-0.69, 2.87) p =0.23 |
| Religious: Secular | **5.01 (1.88, 8.14) p =0.002** | **5.06 (1.94, 8.18) p =0.002** |
| Year collected: 2020/2021 | **-3.51 (-6.77, -0.25) p =0.03** | **-3.53 (-6.75, -0.31) p =0.03** |
| Year collected: 2021/2022 | 0.49 (-1.52, 2.51) p =0.63 | 0.52 (-1.48, 2.52) p =0.61 |
| Year: 9 | 0.88 (-4.61, 6.37) p =0.75 | 0.68 (-4.78, 6.15) p =0.81 |
| Year: 10 | 0.94 (-6.97, 8.86) p =0.82 | 0.75 (-7.14, 8.64) p =0.85 |

Table C. Pupils’ free sugar intake at lunch, excluding those reporting <400 kcal or >4000 kcal over 24 hours

|  | **Model coefficient with all values included** | **Model coefficient with extreme values excluded** |
| --- | --- | --- |
| Pupils | 1,878 | 1,839 |
| Schools | 36 | 36 |
| SFS Status: Mandated | **-2.78 (-4.66, -0.90) p =0.004** | **-2.29 (-4.07, -0.50) p =0.01** |
| Sex: Male | -0.21 (-2.00, 1.59) p =0.82 | -0.32 (-2.02, 1.39) p =0.72 |
| Sex: Other | 0.36 (-4.63, 5.36) p =0.89 | 0.44 (-4.28, 5.17) p =0.85 |
| Age (years) | -1.13 (-3.67, 1.42) p =0.39 | -2.06 (-4.47, 0.36) p =0.10 |
| Ethnicity: Asian/Asian British | -1.69 (-4.12, 0.74) p =0.17 | -1.24 (-3.54, 1.06) p =0.29 |
| Ethnicity: Black/African/Caribbean/Black British | 1.71 (-2.31, 5.73) p =0.40 | 1.62 (-2.18, 5.42) p =0.40 |
| Ethnicity: Mixed/Multiple | -3.04 (-6.80, 0.73) p =0.11 | -2.87 (-6.47, 0.72) p =0.12 |
| Ethnicity: Other | 2.57 (-2.35, 7.49) p =0.31 | 0.79 (-3.93, 5.50) p =0.74 |
| IMD Quintile: 2 | 2.29 (-0.53, 5.11) p =0.11 | 2.08 (-0.60, 4.77) p =0.13 |
| IMD Quintile: 3 | 1.41 (-1.47, 4.30) p =0.34 | 2.19 (-0.55, 4.93) p =0.12 |
| IMD Quintile: 4 | 0.87 (-2.19, 3.92) p =0.58 | 1.84 (-1.06, 4.74) p =0.21 |
| IMD Quintile: 5 | 0.32 (-2.73, 3.37) p =0.84 | 1.11 (-1.79, 4.01) p =0.45 |
| Source of Lunch: 100% School Provided | **-3.55 (-5.18, -1.91) p < 0.001** | **-3.38 (-4.93, -1.83) p < 0.001** |
| Percent FSM | 0.07 (-0.04, 0.18) p =0.20 | 0.08 (-0.02, 0.18) p =0.12 |
| School Level IDACI | **0.12 (0.02, 0.23) p =0.03** | **0.11 (0.01, 0.21) p =0.04** |
| Sixth Form | **-3.18 (-5.14, -1.21) p =0.002** | **-2.34 (-4.20, -0.48) p =0.01** |
| Provision: External | 1.12 (-0.67, 2.91) p =0.22 | 1.35 (-0.35, 3.05) p =0.12 |
| Religious: Secular | **5.01 (1.88, 8.14) p =0.002** | **4.62 (1.65, 7.58) p =0.002** |
| Year collected: 2020/2021 | **-3.51 (-6.77, -0.25) p =0.03** | **-2.81 (-5.90, 0.29) p =0.08** |
| Year collected: 2021/2022 | 0.49 (-1.52, 2.51) p =0.63 | 0.59 (-1.32, 2.50) p =0.54 |
| Year: 9 | 0.88 (-4.61, 6.37) p =0.75 | 3.22 (-1.98, 8.42) p =0.22 |
| Year: 10 | 0.94 (-6.97, 8.86) p =0.82 | 3.52 (-3.99, 11.03) p =0.36 |
